# Studying the characteristics of nanobody CDR regions based on sequence analysis in combination with 3D structures

**DOI:** 10.1186/s43141-022-00439-9

**Published:** 2022-11-21

**Authors:** Tuom Thi Tinh Truong, Viet Quoc Huynh, Nam Tri Vo, Hoang Duc Nguyen

**Affiliations:** 1 Center for Bioscience and Biotechnology, University of Science, Ho Chi Minh City, Vietnam; 2 Vietnam National University, Ho Chi Minh City, Vietnam; 3 Cancer Research Laboratory, University of Science, Ho Chi Minh City, Vietnam; 4 Laboratory of Molecular Biotechnology, University of Science, Ho Chi Minh City, Vietnam

**Keywords:** Nanobody, CDR1, CDR2, CDR3, Parameters

## Abstract

**Background:**

Single-domain antibodies or nanobodies have recently attracted much attention in research and applications because of their great potential and advantage over conventional antibodies. However, isolation of candidate nanobodies in the lab has been costly and time-consuming. Screening of leading nanobody candidates through synthetic libraries is a promising alternative, but it requires prior knowledge to control the diversity of the complementarity-determining regions (CDRs) while still maintaining functionality. In this work, we identified sequence characteristics that could contribute to nanobody functionality by analyzing three datasets, CDR1, CDR2, and CDR3.

**Results:**

By classification of amino acids based on physicochemical properties, we found that two different amino acid groups were sufficient for CDRs. The nonpolar group accounted for half of the total amino acid composition in these sequences. Observation of the highest occurrence of each amino acid revealed that the usage of some important amino acids such as tyrosine and serine was highly correlated with the length of the CDR3. Amino acid repeat motifs were also under-represented and highly restricted as 3-mers. Inspecting the crystallographic data also demonstrated conservation in structural coordinates of dominant amino acids such as methionine, isoleucine, valine, threonine, and tyrosine and certain positions in the CDR1, CDR2, and CDR3 sequences.

**Conclusions:**

We identified sequence characteristics that contributed to functional nanobodies including amino acid groups, the occurrence of each kind of amino acids, and repeat patterns. These results provide a simple set of rules to make it easier to generate desired candidates by computational means; also, they can be used as a reference to evaluate synthetic nanobodies.

**Supplementary Information:**

The online version contains supplementary material available at 10.1186/s43141-022-00439-9.

## Background

Antibodies (Abs), as well-known human therapeutic options, have been widely adopted and used for treating numerous diseases. High-throughput screening (HTS) proved to be an efficient approach to screen for lead Abs that bind to specific antigens [3, 7, 43]. However, these methods were labor- and resource-intensive and time-consuming and required advanced laboratory skills. With the exponential growth in the amount of information about compounds and molecules that have high potential in clinical and industrial uses, the development and application of in silico or virtual screening have been encouraged to overcome the costs of HTS. This new approach involves “implement rooted in physical principles and/or experimental knowledge to prioritize compounds for experimental testing, thus aiming to save time and cost through a more rational approach compared to HTS” [33]. Different approaches have been adapted for screening small and large therapeutic molecules. For selecting small molecule candidates, in silico screening methods incorporate simple filtering based on physicochemical properties such as Lipinski’s rule of 5 [25] or more complex methods like docking-based algorithms, which mimic the interaction between a drug molecule and its target. Lipinski’s rule of 5 (Ro5), a robust guideline for screening oral drug-like compounds, has been widely used by medicinal and computational chemists. The rule defines a set of cutoff values that can be used to screen for molecules whose properties violate the boundary values and therefore would be less suitable for oral use. Because of its simplicity and convenient application, many related studies have used this method, which led to the development of the in silico screening strategy commonly used in pharmaceutical research. In case of large immunoglobulin molecules like Abs, most of the methods that were used to develop Ab candidates involved analyzing and engineering the variable region and Fc region. Many studies have focused on the variable region because it is not only responsible for the antigen binding ability but also affects the pharmacokinetics, pharmaceutical properties, and immunogenicity of Abs [15]. The analysis of the variable region comprises predicting potential physicochemical degradation sites, immunogenicity, and aggregation [23] with the help of computer algorithms. The engineering of the variable region includes changes in antigen-binding-site properties, pharmacokinetics, pharmaceutical properties, and immunogenicity. However, these methods are complicated and require good computational resources to screen for potential candidates.

With the increasing use of nanobodies (Nbs), recombinant single-domain variable fragments of camelid heavy chain-only Abs, the field of therapeutic molecules has become more competitive. Nbs have superior properties for medical diagnosis and therapeutic applications due to their high affinity, high production yield in a variety of expression systems, small size, high stability, and solubility together with the ability to recognize unique epitopes that traditional Abs cannot [13]. Nbs have been used in protein purification [21] and immunoprecipitation [29] and as crystallization-assisting chaperones [22]. In addition, Nbs can be used in the clinical field as bioimaging tools [35], for disease diagnosis [40], targeting therapeutics [14], identifying protein-protein interaction, and much more. With the current COVID-19 pandemic still ongoing in many parts of the world, the potential of Nbs has been demonstrated for detecting and treating COVID infection [2, 45, 47]. Although antiviral drugs are also solid options for treating virus infections, the process of screening and random trial controls would generally hamper the adoption of anti-COVID drugs [46]. Isolated Nbs could also be effective in solving the global problems of COVID variants [11, 41, 50], which have been challenging vaccine efficacy. The demand has driven researchers to produce functional Nbs against COVID-19 [38, 44, 49]. Given the great potential for nanobody applications, detailed information on their formation and use has become essential for researchers. Multiple methods have been developed and utilized for screening the best Ab [5, 24, 28, 39] and Nb candidates [10, 12, 30] for therapeutic properties. Currently, synthetic immunoreactive molecules can be obtained through the screening of appropriate libraries based on structure or sequence [37]. However, no such software for Nbs has been developed. The bottleneck in finding candidate Nbs is to overcome the enormous sequence diversity, because only a small fraction of them may be functional. Research has been conducted to design a library with only four-amino acid codes to reduce the diversity in the Nb sequences [9] or, with the help of computational methods such as SwiftLib, to limit the threshold of diversity [16]. Prior knowledge of functional CDRs could also be used to graft CDR sequences onto Nb frameworks to generate Nbs that are difficult to make by traditional means [42]. The combination of both sequence characteristics and structures generated by modeling software such as Rosetta [17] or AlphaFold [18] can significantly speed up the discovery of therapeutic Nbs. In order to do that, it is necessary to analyze Nb sequences to define characteristics that can be used for library design or to create Nbs with desired functions and stability.

Here, we have studied a large number of CDR sequences of Nbs to find their overall sequence characteristics and the specified constraints that could be present in the CDR loops of known Nbs. We also analyzed 3D structural features in combination with primary sequence data to explain some definitive characteristics of the functional Nbs.

## Methods

### Creation of the nanobody CDR database

To construct the Nb CDR datasets which are used for analysis, we first downloaded all Nb sequences from the NCBI database. We used ANARCI [8] with the Martin [1] numbering scheme to number the downloaded sequences and filtered out sequences that were not immunoglobulin molecules. We then removed Abs and Ab variations such as Fab and scFv from the Nb dataset based on the sequence description. The amino acid positions spanned the regions from 30 to 35, 47 to 58, and 93 to 101 of CDR1, CDR2, and CDR3, respectively.

### Amino acid group classification

We classified all 20 kinds of amino acids into four groups based on their side-chain properties [20]: (1) nonpolar amino acids, G, A, V, L, I, P, and M; (2) polar-neutral amino acids, S, T, C, N, and Q; (3) electrically charged amino acids, E, D, K, R, and H; and (4) aromatic amino acids, F, Y, and W. For each CDR sequence, we assigned each amino acid to its corresponding group, counted the number of AAs in each group, and expressed this as a percentage of the total. For instance, the sequence YVGG can be designated “4111,” which shows that it contains two groups: group 1, which accounts for 75%, and group 4 which makes up 25% of the total residues.

### Highest possible counts of amino acids in CDR sequences

For each CDR sequence, we extracted the highest number of each kind of amino acids (except cysteine) and the respective length of that sequence. Scatter plots were used to analyze the possible correlation between the highest possible count of a certain kind of amino acid and all the observed CDR sequence lengths.

### Tandem single amino acid and oligopeptide repeats

For tandem single amino acid repeats, a minimum length of at least three consecutive single amino acids was considered. For tandem oligopeptide repeats, a minimum length of two, containing at least two different amino acids, was applied. We analyzed each sequence and observed the count of single amino acid/oligopeptide repeats and their lengths in correlation with the sequence length. Due to the high diversity in the amino acid composition of CDRs, only oligopeptide tandems with lengths of two or three were used for the analysis.

### Visualization of conserved dominant amino acids in crystal structures

Antigen-free Nb 3D structures were extracted from the RCSB protein database (PDB ID: 5M2W, 6OBC, 7KJH, 4WEU, 6Z20, 6OBM). Wincoot with SSM Superpose function was used to superimpose extracted Nb 3D structures. For PDB entries with duplicate Nb chains, redundancy was checked, and only one chain was retained. We identified the positions in each CDR that had structurally conserved AAs by calculating the frequency of the most dominant AAs. Each highly conserved position in each CDR was visualized in 3D by using PyMOL. For analyzing the interaction between a Nb and antigen (PDB ID: 7KGJ), we numbered the Nb sequence by using Martin’s scheme to highlight the specific CDR positions that could interact with the antigen or were structurally conserved. Investigation of P at position H96 was done with three superimposed Nbs (PDB ID: 3K7U, 6EY0, 6QGW).

## Results

### Length variation and overall amino acid composition in nanobody CDRs

**Fig. 1:**
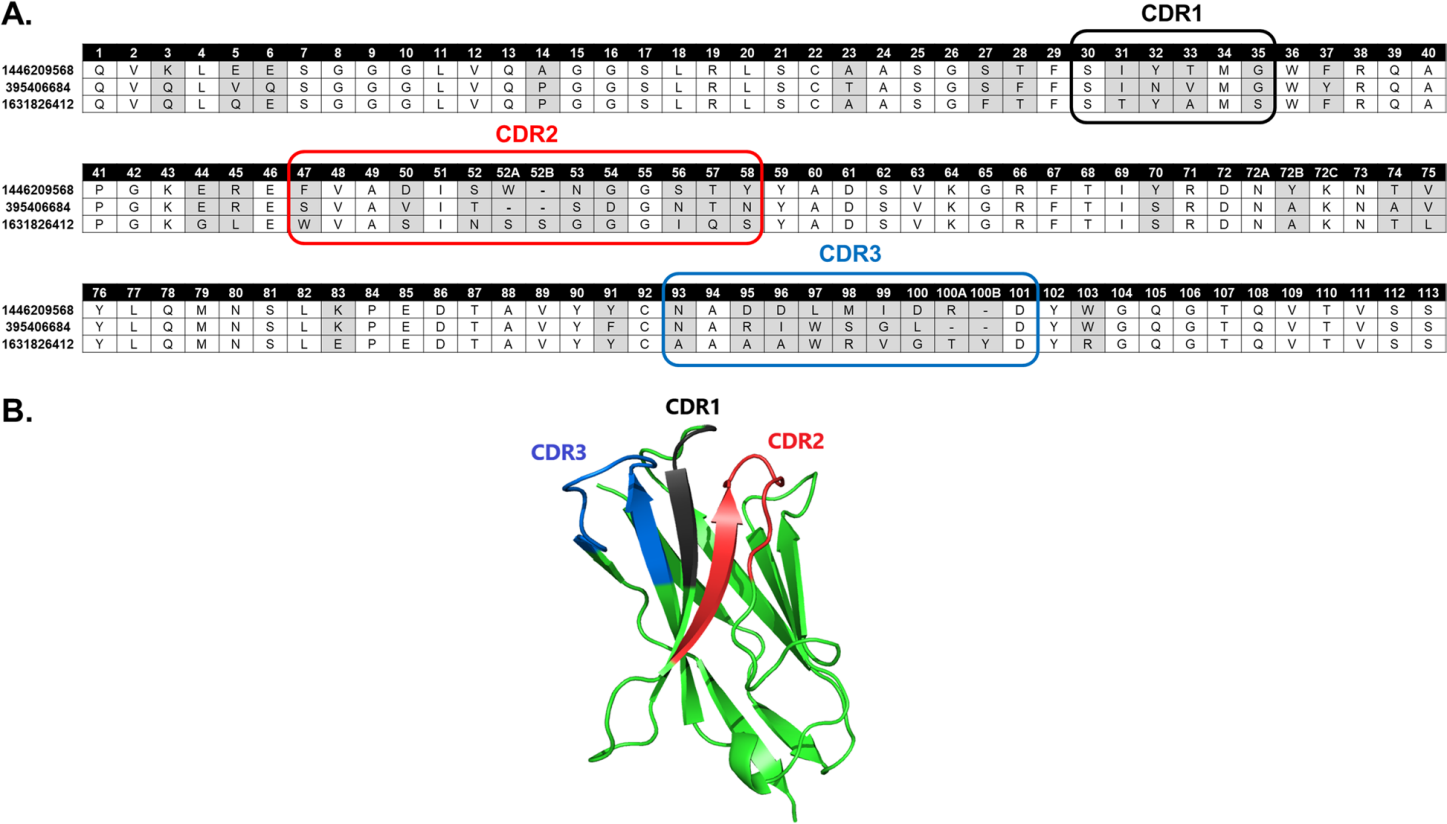
CDRs of Nbs. **A** Representative Nb sequences with CDR1, CDR2, and CDR3 separated by conserved frameworks; residue positions were numbered by the widely used Martin scheme [1]. The Martin numbering scheme can accurately define the position of residues in the Nb sequences, which can then be used to locate the CDR automatically and conveniently. Positions with different residue alignment were colored in grey. These Nbs interacted with internalin B (gi: 1446209568, pdb: 6DBE), KRT19 (gi: 395406684), and melanoma-associated antigen B1 (gi: 1631826412, pdb: 6R7T), respectively. **B** CDR1, CDR2, and CDR3 loops in a crystallized structure

**Fig. 2:**
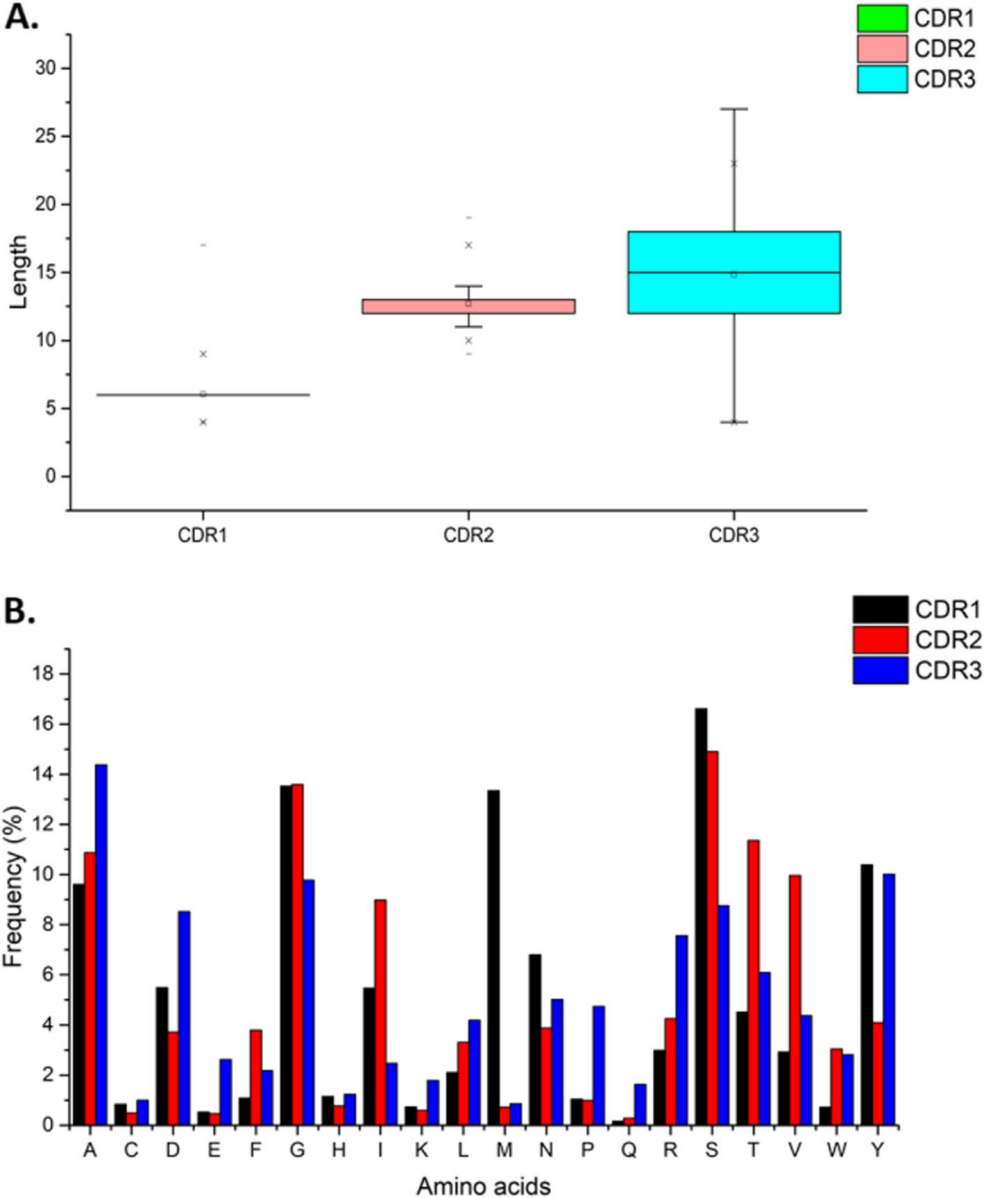
Distribution of sequence lengths and amino acid composition of CDRs from Nbs. **A** CDR length. **B** Amino acid composition

Nbs can interact with antigen mainly by CDR3, but also by CDR1 and CDR2. These three regions are separated by conserved frameworks (Fig. 1). We downloaded and processed 2161 non-redundant and numbered Nbs from NCBI, resulting in 2377 CDR1, 2377 CDR2, and 2380 CDR3 sequences. The distribution of sequence length varied with each type of CDR, and the optimal CDR length increased from CDR1 to CDR3. CDR1 and CDR2 had consistently average lengths with six residues in CDR1 and 12–13 in CDR2. However, the CDR3 showed large variations in sequence length, with values ranging from 12 to 18 and a median value of 15 (Fig. 2A). After analyzing the frequency of AAs, we also found differences in the composition of widely used residues in each CDR. Methionine was mostly found in the CDR1 (> 13% of sequences) and rarely found in CDR2 and CDR3 (< 1%), while P was more frequent in CDR3 (~ 5%), compared with CDR1 or CDR2 (~ 1%) (Fig. 2B).

### Frequency of amino acid groups in CDR sequences

**Fig. 3:**
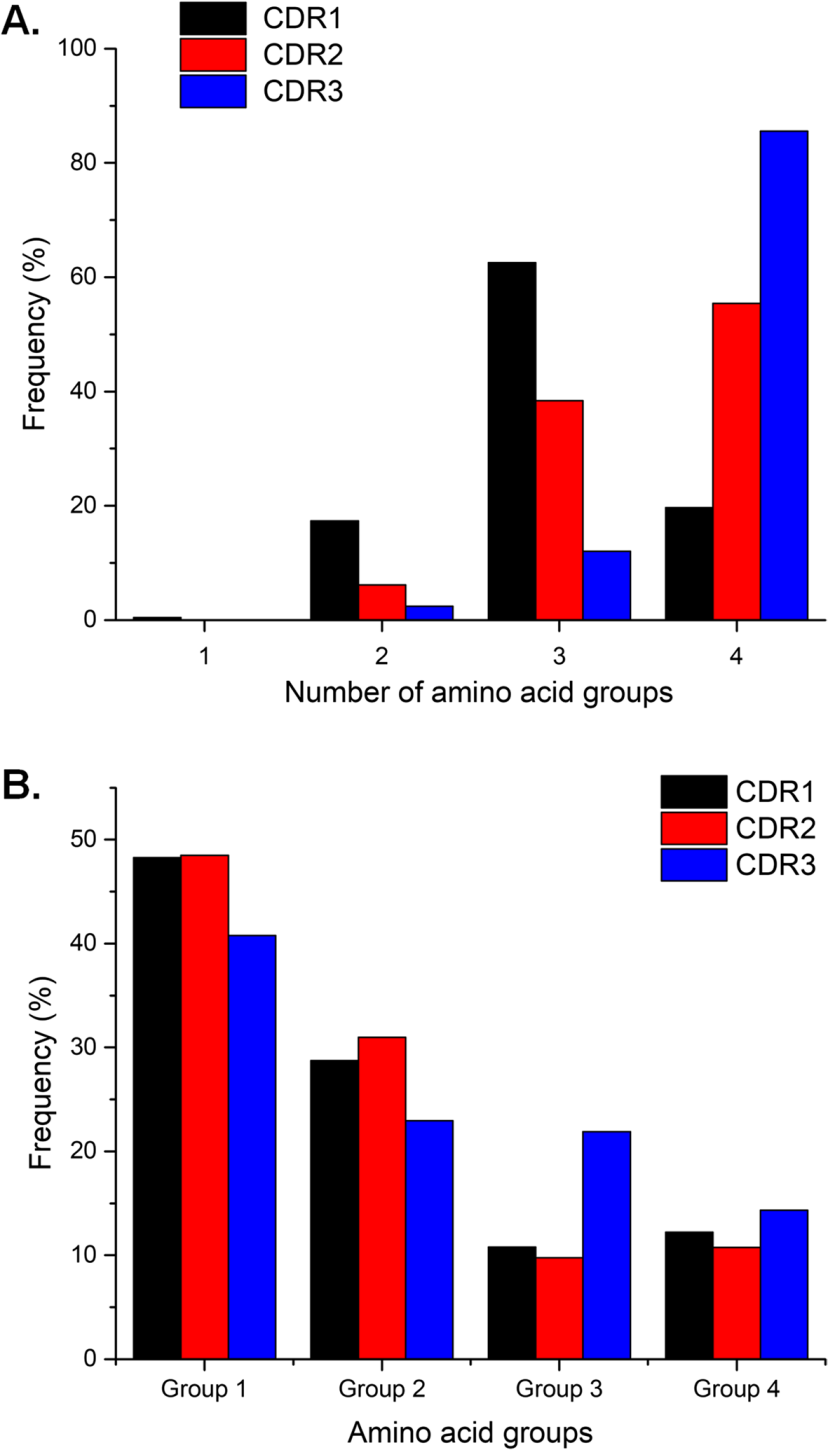
Frequency of the total number of AA groups (**A**) and frequency of each AA group in CDRs of Nbs (**B**)

We first investigated whether the contribution of different kinds of AA groups reflected their roles in CDRs. The frequency of different AA groups showed some interesting features. All Nb CDRs required AAs from at least two different groups in their sequences (Fig. 3A). The AAs were classified based on their side-chain properties, into nonpolar, polar-neutral, electrically charged, and aromatic. However, CDR1 had significantly lower diversity in AA groups compared to CDR2 and CDR3. The majority of CDR1 sequences contained three AA groups (63%), while most of the CDR2 and CDR3 sequences contained all four AA groups (55% and 86%, respectively) (Fig. 3). This might indicate that all CDR loops contain various functional AA groups to modulate their backbone conformation and their affinity upon interacting with antigens. Most CDR1 sequences need only two different AA groups, while CDR2 sequences need more than two, and CDR3 sequences need all four different groups to achieve functionality.

The frequency of AA group usage also differed among the types of CDR (Fig. 3B). Interestingly, CDR1 and CDR2 loops shared similar frequencies in the usage of all four AA groups. Group 3 AAs were most common in CDR3 (22%), compared to CDR1 (11%) and CDR2 (10%).

### Limitations in the distribution of amino acids in CDRs

**Fig. 4:**
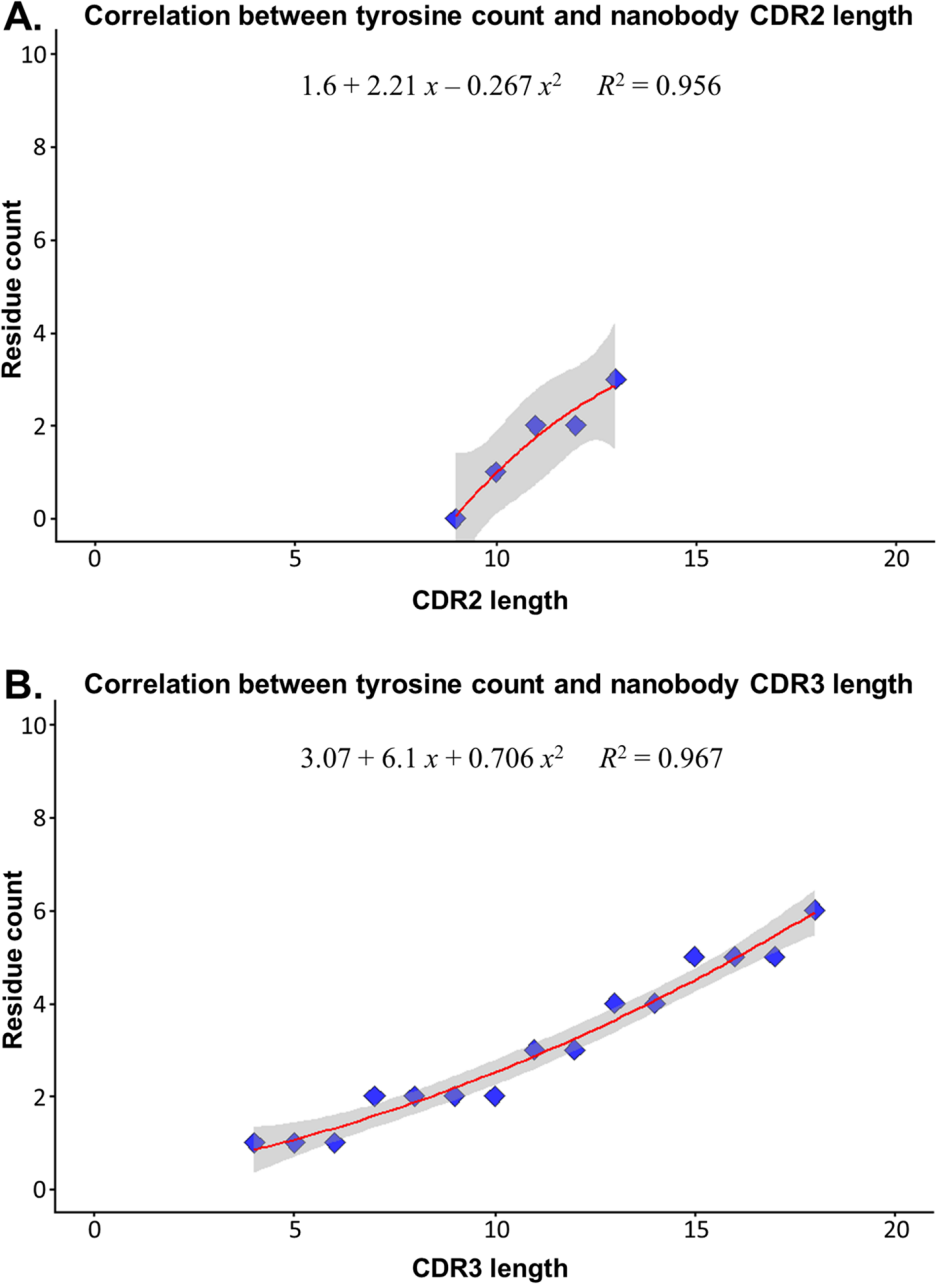
Correlation between the highest occurrence of tyrosine and CDR lengths. **A** CDR2. **B** CDR3

The composition of AAs in CDR sequences is naturally highly diverse and randomized. However, constraints on the use of certain AAs could potentially exist to prevent a CDR from being non-functional. To determine which AAs might be limited in a CDR and whether the selection followed any trend, we determined the greatest occurrence of each group in relation to the lengths of CDR loops. The results showed a strong correlation between CDR length and the abundance of specific types of AAs. For simplicity and practical reasons, we used a threshold length of 13 for CDR2 and 18 for CDR3 based on the observed length distribution (Fig. 2A). The results indicated that the frequency trends in CDR2 and 3 were consistent and could be represented by polynomial equations. For example, a quadratic equation could describe the highest occurrence of tyrosine (Fig. 4). The highest occurrence of each AA type was more consistent in the case of CDR2 and less consistent in CDR3. For the CDR2 loops, most of the residues showed a strong correlation with *R*^2^ > 0.8 (except for G and P) (Supplement 1). For the CDR3 loops, AAs D, S, and Y showed a high correlation with *R*^2^ values of 0.837, 0.93, and 0.967, respectively. However, other AAs in the CDR3 showed lower correlation values (Supplement 2). The CDR1 was excluded as this region was mainly restricted to a length of six AAs.

### Amino acid repeat units rarely present in CDRs

**Fig. 5:**
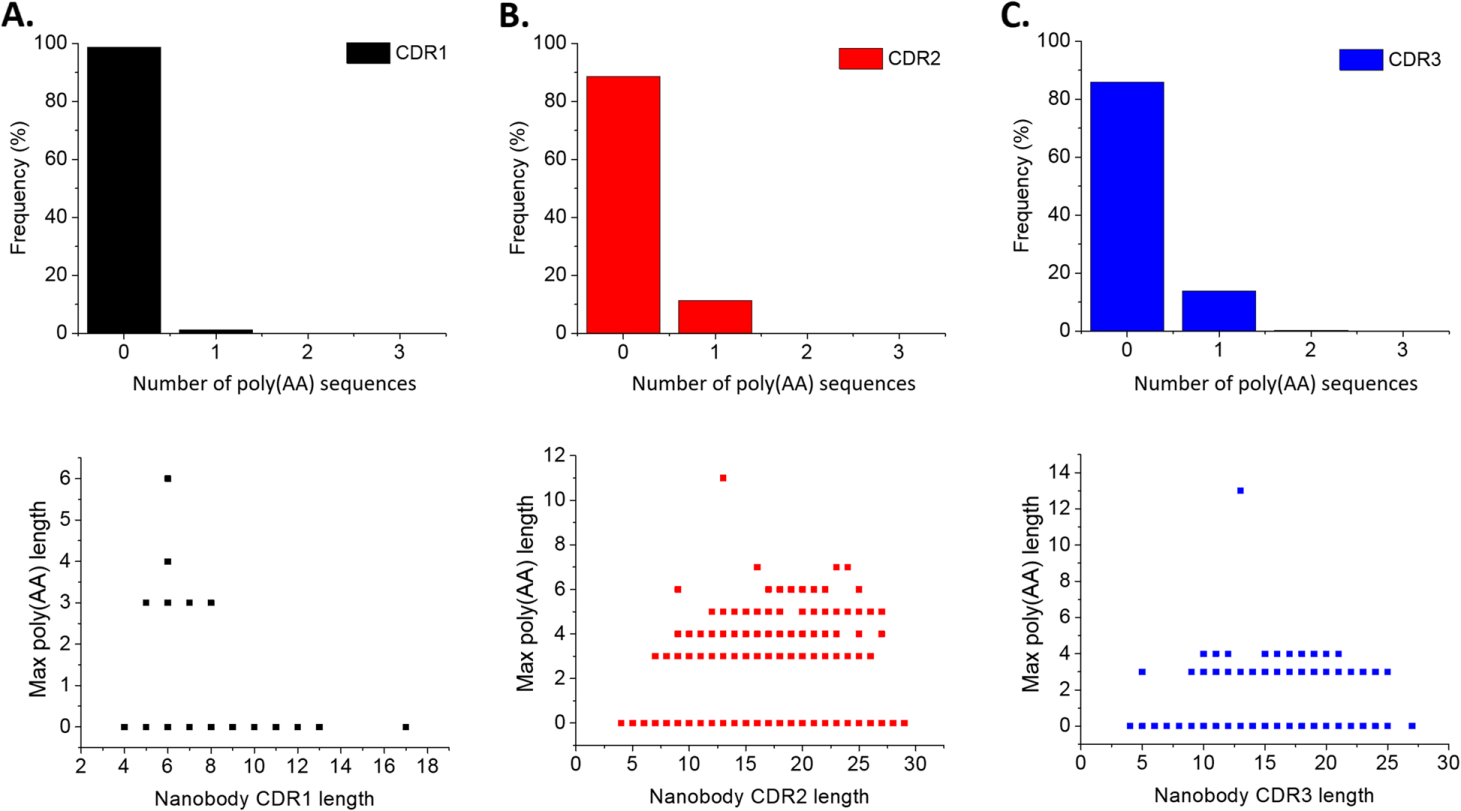
Distribution of polyamino acids and correlation between their occurrence and CDR length. **A** CDR1; **B** CDR2; **C** CDR3

After investigating the composition and presence of each AA, we then analyzed more complex sequence features like repeating units in the CDRs of Nbs to determine the frequency of repeated AA sequences in CDRs of functional Nbs. We first identified polyamino acid, poly(AA), repeats with length > 3, and the results indicated that the frequency of such repeats was limited (Fig. 5). Poly(AA) stretches were rare in most CDR sequences and accounted for only 1.26%, 11.36%, and 14.12% of CDR1, CDR2, and CDR3 sequences, respectively. The presence of poly(AA) repeats was constrained to one unit per CDR, with only one exception, where one out of 2380 Nb CDR3s harbored three different poly(AA) stretches (gi 1036392491). The lengths of poly(AA) repeats did not correlate with CDR length. Base on the scatterplots, only CDR2 showed a small correlation with the length of poly(AA) repeats (Fig. 5).

Oligopeptide repeats were also under-represented in Nb CDRs (Table 1). The 2-mer repeat sequences were under-represented, as only 16 (0.67%) CDR1, 75 (3.16%) CDR2, and 148 (6.2%) CDR3 harbored 2-mer repeat sequences. Furthermore, the length of this kind of repeat was mostly limited to four residues. Not all kinds of AAs can form 2-mer repeats, as observed. In CDR2, G*, S*, and T* (* stands for any AAs that form a 2-mer with the preceding residues) accounted for more than 90% of 2-mer repeats (Supplement 3A). In CDR3, the composition of 2-mer repeats was more widely distributed over different kinds of AAs; Y* (24.34%), L* (15.13%), and S* (11.84%) were the most common 2-mer repeats (Supplement 3B). The number of 3-mer tandem repeats was also highly restricted in the CDRs, as only 3 (0.13%), 9 (0.38%), and 6 (0.25%) of CDR1, CDR2, and CDR3 loops contained 3-mer repeats.

**Table 1:** Frequency of oligopeptide repeats in the CDR sequences of Nbs

	CDR1 (*n* = 2377)	CDR2 (*n* = 2377)	CDR3 (*n* = 2380)
2-mer repeats	16 (0.67%)	75 (3.16%)	148 (6.2%)
3-mer repeats	3 (0.13%)	9 (0.38%)	6 (0.25%)

### Structurally conserved amino acids in CDR sequences

**Fig. 6:**
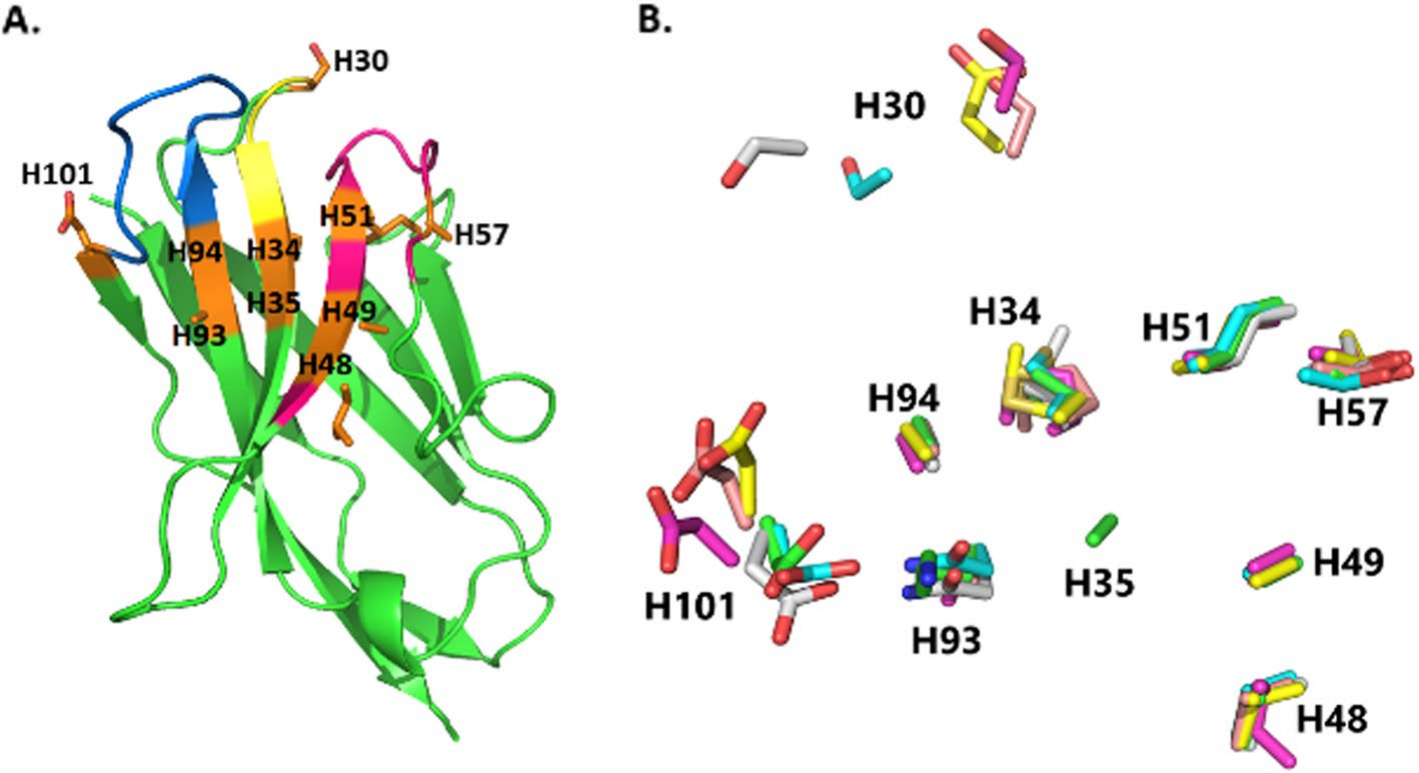
Key CDR positions of a Nb. **A** Crystal structure of a Nb with highlighted positions, yellow: CDR1, red: CDR2, blue: CDR3. **B** Side-chain coordination of six superimposed Nb structures. Each numbered position could comprise more than one kind of amino acid. H30: 4 S, 1 D, 1 G; H34: 6 M; H35: 5 G, 1 A; H48: 5V, 1L; H49: 6A; H51: 6I, H57: 5T, 1A; H93: 3A, 2N, 1K; H94: 6A; H101: 6D

After investigating the differences in the sequence characteristics between CDRs, we explored the possible conservation of specific AAs at numbered CDR position based on their frequencies. We chose ten positions that tended to contain a certain kind of AA: H30, H34, and H35 in CDR1; H48, H49, H51, and H57 in CDR2; H93, H94, and H101 in CDR3 (Fig. 6). These positions have been shown to be dominated by specific AAs. At position H30, S was found to have the highest frequency (64.3% of the total CDR1 dataset), but their side-chain coordination varied greatly. M was found to be mostly enriched in the CDR1 sequences, especially at position H34 (77.4% of the total CDR1 dataset) with overlapping of side-chain coordination. G was conserved at position H35 (67.4% of the total CDR1 dataset), followed by A (15.3%). For the CDR2 dataset, V and A were predominant at positions H48 and H49 (96.6% and 75.4%, respectively) and showed high concordance in side-chain coordination. At positions H51 and H57, I and T were highly conserved (79.9% and 75.1%, respectively), and their side-chain conformation overlapped strongly. For the CDR3 dataset, position H93 showed a high frequency of A and N (58.9% and 17.2%, respectively) with a high degree of overlapping conformation. Interestingly, position H94 was dominated by A (66.8%). H101 showed high conservation of D (50.5%), but the side-chain coordination also varied significantly between 3D structures.

**Fig. 7:**
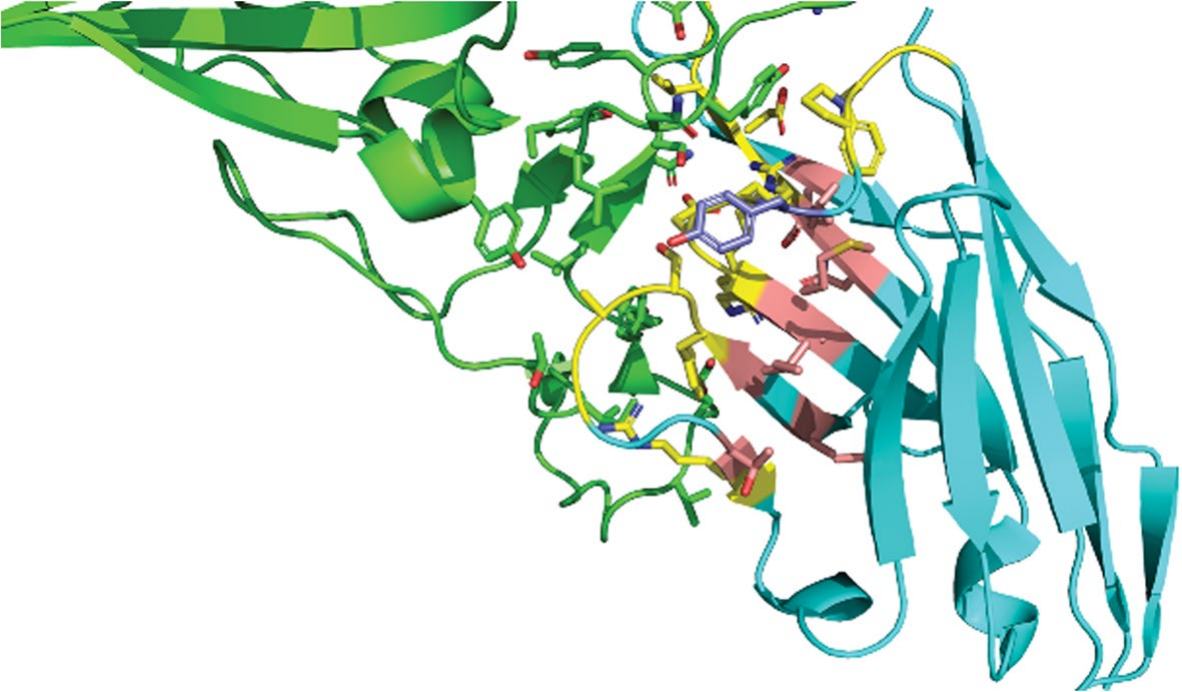
A complex of a Nb and SARS-CoV-2 receptor-binding domain (RBD) (PDB ID: 7KGJ). The side chains of all residues that participated in the interaction are shown. RBD is green, and Nb sb45 is cyan. Residues of the Nb interacting with the antigen are yellow. Residues in the conserved CDR position are light pink. Residues in the conserved CDR position interacting with antigen are purple

We inspected another complex, and this one was between the synthetic Nb, Sb45, and the SARS-CoV-2 receptor-binding domain (PDB ID: 7KGJ) (Fig. 7). In this structure, the Nb interacted with the spike glycoprotein through all three CDRs. The marked residues from the Sb45 Nb were translated to the specific positions by using Martin’s numbering scheme. The length of CDR1 was six AAs, CDR2 was 13 AAs, and CDR3 was 13 AAs. Out of ten observed positions with high AA conservation, nine positions (light pink) mainly contributed to beta-sheet formation rather than directly interacting with antigen residues. However, the residue T (purple) at position H30 (dominated by S) interacted with the antigen. The presence of AAs at positions H34, H35, H48, H49, H51, H57, H93, and H101 was also concordant with the dominant residues. Examination of sequence characteristics revealed no poly(AA) stretches comprised of three or more AAs or any oligopeptide repeats.

**Fig. 8:**
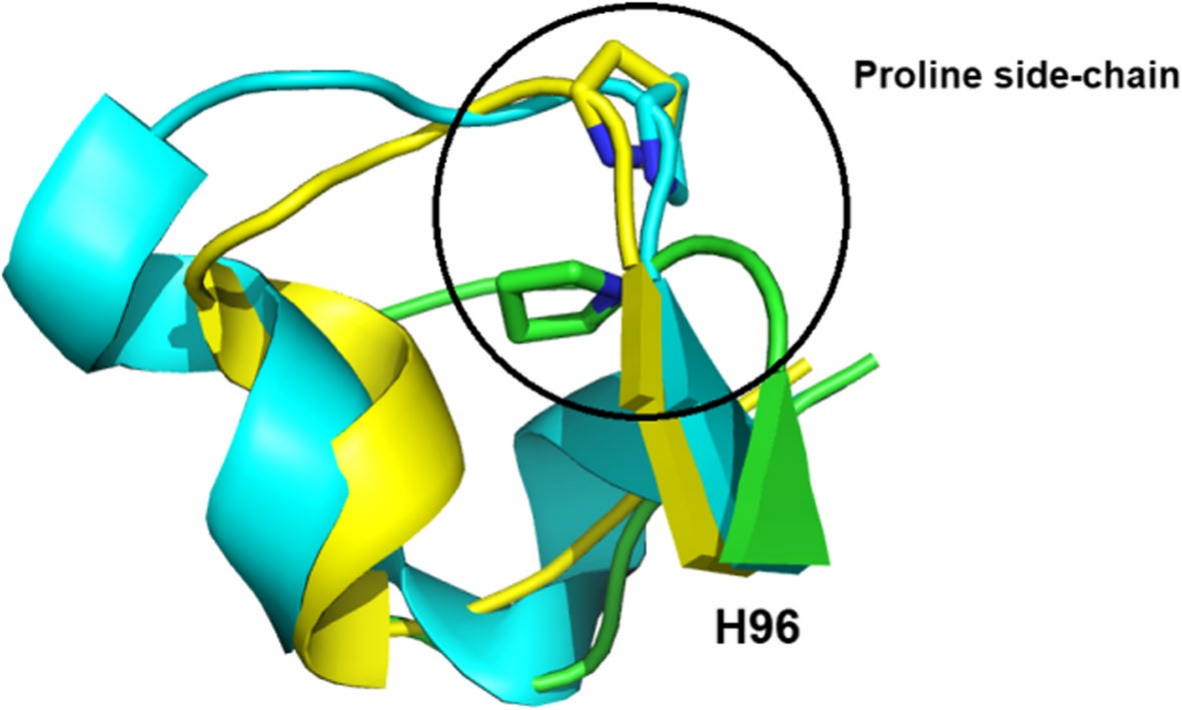
CDR3 loops from three superimposed Nbs (PDB ID: 3K7U, 6EY0, 6QGW). The proline side chain at H96 is shown as a ring

Notably, P was mainly found in the CDR3 (Fig. 2B) and was located at position H96 (10.3%). We inspected three crystal structures (PDB ID: 3K7U, 6EY0, 6QGW) with P at the H96 position. The data revealed that all three CDR3 loops had a sharp bend through P, which verified the role of proline residues in maintaining CDR3 loop conformation (Fig. 8).

## Discussion

In this study, we analyzed 2377 CDR1, 2377 CDR2, and 2380 CDR3 Nb sequences to reveal their characteristics. We first analyzed the CDR loop length because many studies have suggested that length variation may relate to the diversity and AA composition [6, 34, 48]. The variation in length was greater between CDRs. CDR1 was the shortest with an optimal length of six AAs; CDR2 and CDR3 were much longer with a broader range of optimal length. CDR3 was the longest perhaps to compensate for the lack of a VL to maintain sufficient binding surface area [27]. Thus, choosing an appropriate length should be considered for Ab or Nb CDR engineering.

After establishing the optimal CDR length, we then proceeded to investigate the diversity and AA composition. The usage of total AAs in the CDR (Fig. 2) was analyzed to gain insight into how each CDR differed from the other. Some AAs were more common in one CDR but less so in others. Methionine, for example, had a high frequency in CDR1 but was rarely found in CDR2 or CDR3. Glutamine and proline were present at a higher level in CDR3 than CDR1 or CDR2. These results suggest that certain kinds of AA might be more favorable in specific CDRs while less essential AAs could be discarded from the CDR sequence.

Due to the high diversity in AA composition in the CDR sequences, it is essential to classify AAs according to their side-chain structure to identify the general characteristics. The AA composition in the CDR sequences was so diverse and hard to predict, that they still maintained a combination of at least two different AA groups (Fig. 3). Because AAs are the critical element determining loop conformations and specificity [36], Nbs should adopt a set of residues with specific physicochemical properties so that their CDR loops can function correctly. For example, an aromatic residue like Y contributes significantly to specific interactions and affinity [9]; however, its side chain is large and contains a hydrophobic ring. A loop full of aromatic residues would result in a rigid conformation that was highly aggregated because of the tendency for π-stacking [4]. These CDR loops must incorporate some smaller, more flexible AAs like A and S in their sequences to provide better backbone flexibility and for appropriate positioning of the aromatic side chains [9]. In contrast, polar and charged residues contribute to better solubility [31, 32]. It is worth noticing that CDR3 contained more different groups of AAs than the other two CDRs. This suggests that CDR3 may require different AAs for optimal function to compensate for the monomeric Nb form, as CDR3 has longer loops and a larger interface for optimal antigen interaction.

When the general characteristic of AA groups in the CDR was identified, we then delved further into the individual AAs to explore their properties in the CDR loops. We suspected that although the AA composition in this region was diverse, there could be a constraint in the occurrence of each kind of residue. For example, a CDR3 loop that contained a stretch of only one kind of AA should not exist because the loop would not be able to function properly and the chances of forming a long poly(AA) stretch were statistically very small. In this regard, the balance in AA composition is fundamental, and this feature should be correlated with the length of the CDR loops. To check whether this hypothesis was true, we calculated the highest occurrence of each kind of residue in each CDR. The results that we found were promising, as many AAs showed an increasing trend in their correlation with the length of CDR loops, but the trends of the AAs as a whole were not always consistent. Only some residues showed high correlation values, and these residues were also abundant in the CDR AA composition; low-frequency residues showed low correlations. We suspected that these favored AAs were optimized because of their major roles in the CDRs, while others were less optimized because of their under-representation and minor contribution to CDR function, thus resulting in the variability in trends. However, only some AAs indicated a clear rising trend. This inconsistent finding might be related to the limited Nb data that we retrieved, as only about 2400 Nb sequences were obtained. More data about functional Nbs should become available in the near future so we can determine whether our hypothesis about the occurrence of AAs is also consistent with the Nb case. Once the distribution of each kind of AA in the CDR loops is completely established, it will significantly contribute to the application of Ab-Nb CDR engineering by identifying the array of AAs that balances the AA composition.

After analyzing the AA composition, we next studied AA repeat patterns in the CDR sequences. AA repeats are abundant and have particular roles in protein function [19, 26], so we checked whether these features also applied to the CDR sequences of Nbs. We first studied the characteristics and distribution of poly(AA) stretches with a minimum length of three. We found that there was a constraint in the occurrence of poly(AA) stretches in the CDR loops, where three was the most common length for poly(AA)s. The frequency of occurrence values was also low as most of the Nbs’ CDR loops did not contain any poly(AA) tracts. Not all kinds of AAs can form poly(AA) tracts, and only a portion of them significantly contributed to poly(AA) formation. This result may be related to the usage of AAs in each CDR, where only abundant AA kinds had a higher chance of forming these poly(AA) tracts. The average length of CDR1 was significantly shorter than that of CDR2 and CDR3, which explained the rarity of occurrence of poly(AA) in CDR1 compared to CDR2 or CDR3. In the case of oligopeptide repeats, the result showed a high restriction in the occurrence of repeat units. In the CDR2, most 2-mer repeats were formed by common AAs such as G, S, and T. In CDR3, the composition of 2-mers was much more varied than in CDR2, which could be related to the innately high diversity in CDR3. Almost all of the dipeptide repeats were constrained to a length of four (two repeats). This observation was also applied to tripeptide repeats in which the greatest length was six (two repeats). Nbs could not be functional as expected if the CDRs contained too many repeats, even though the AA composition and AA groups satisfied observed parameters. We found that protein repeats and poly(AA) tracts were highly under-represented in the CDRs, implying that these repeating patterns played only a minor role in the CDR loops.

With the increasing interests in both Ab and Nb engineering, it is important to identify the AAs that can reasonably be modified. Many studies have been conducted to select the potential AA candidates at certain positions, such as altering the binding properties [37] or improving solubility without affecting binding affinity [31]. The desired positions across different studies should also be carefully checked since they may vary because of differences in the numbering schemes used. By inspecting the 3D crystal structures of six representative Nbs, we found that the conservation between dominant AAs and their spatial coordination was not strictly correlated. We found that AAs at the two ends of CDR loops, such as S at H30 or D at H101, showed greater side-chain coordination diversity than residues inside the CDR loops. We suggested that AAs at the terminal positions of the CDR loops had to be more dynamic and flexible for repositioning the CDR loop conformation, while others inside the loops had to be more rigid to maintain CDR loop stability. In case of M, this kind of AA was mostly found in CDR1 with high conservation at position H34 and overlapping of side-chain coordination. This indicated that M is very selective and well-conserved, implying M may play a different role in the structural conformation of CDR1 rather than interacting directly with antigens. Other AAs at CDR2 such as V at H48, A at H49, I at H51, and T at H57 also showed high frequency and maintained side-chain coordination, which implied major roles in CDR2 loop conformations. Interestingly, A dominated at the position of H94 in Nbs, which is totally different from the same position in Abs (data not shown). Many studies have demonstrated the hallmark AAs at the interface of light chains and heavy chains which can distinguish Nbs from Abs; however, we still have not found the explanation for this distinctive feature of Nbs at the H94 position. Examining a candidate Nb-antigen structure also showed the relationship between well-conserved CDR positions and 3D structure, as AAs at these positions mostly contributed to Nb stability. Some Nbs with a rigid AA, such as P at position H96, could also play a role in maintaining the loop conformation. Thus, modifying the AAs at these positions could affect a Nb’s structure. These results could be useful for limiting the diversity of certain positions on the CDR loops since the usage and coordination of some AAs were well-established.

## Conclusions

The increasing number of Nb sequences has provided useful information about the sequence-based characteristics of the CDR loops, which can be used to define functional Nbs. By extracting and analyzing this data, we found that the presence of two different AA groups was sufficient for Nb CDRs, given the limitation in the occurrence of certain AAs in correlation with CDR lengths and the restriction in AA repeats in the Nb CDRs. This knowledge should be helpful to establish parameters or cutoffs for setting simple rules for generating desired candidate Nbs, particularly in CDR engineering, library designs, or evaluating synthetic Nbs via in silico high-throughput screening.

## Supplementary Information

**Additional file 1: Figure S1.** For the CDR2 loops, most of the residues showed strong correlation with *R*^2^ > 0.8 (except for G and P). **Figure S2.** Correlation between the highest occurrence of amino acids and CDR3 lengths. **Figure S3.** Occurrence of repeat motifs formed by corresponding amino acids in each CDR region. (A) poly amino acid repeats, (B) oligo repeats.

## Data Availability

The nanobody sequence data analyzed in this study is available and was taken from the NCBI protein database on February 24, 2021.

## Abbreviations

AAs: Amino acids
Abs: Antibodies
CDRs: Complementarity-determining regions
HTS: High-throughput screening
Nbs: Nanobodies

## References

[1] Abhinandan KR, Martin ACR (2008). Analysis and improvements to Kabat and structurally correct numbering of antibody variable domains. Mol Immunol.

[2] Anderson GP, Liu JL, Esparza TJ, Voelker BT, Hofmann ER, Goldman ER (2021). Single-domain antibodies for the detection of SARS-CoV-2 nucleocapsid protein. Anal Chem.

[3] Ayriss J, Woods T, Bradbury A, Pavlik P (2007). High-throughput screening of single-chain antibodies using multiplexed flow cytometry. J Proteome Res.

[4] Bemporad F, Taddei N, Stefani M, Chiti F (2006). Assessing the role of aromatic residues in the amyloid aggregation of human muscle acylphosphatase. Protein Sci Publ Protein Soc.

[5] Birch JR, Racher AJ (2006). Antibody production. Adv Drug Deliv Rev.

[6] Collis AVJ, Brouwer AP, Martin ACR (2003). Analysis of the antigen combining site: correlations between length and sequence composition of the hypervariable loops and the nature of the antigen. J Mol Biol.

[7] de Wildt RM, Mundy CR, Gorick BD, Tomlinson IM (2000). Antibody arrays for high-throughput screening of antibody-antigen interactions. Nat Biotechnol.

[8] Dunbar J, Deane CM (2016). ANARCI: antigen receptor numbering and receptor classification. Bioinformatics.

[9] Fellouse FA, Wiesmann C, Sidhu SS (2004). Synthetic antibodies from a four-amino-acid code: a dominant role for tyrosine in antigen recognition. Proc Natl Acad Sci U S A.

[10] Fridy PC, Li Y, Keegan S, Thompson MK, Nudelman I, Scheid JF, Oeffinger M, Nussenzweig MC, Fenyö D, Chait BT, Rout MP (2014). A robust pipeline for rapid production of versatile nanobody repertoires. Nat Methods.

[11] Güttler T, Aksu M, Dickmanns A, Stegmann KM, Gregor K, Rees R, Taxer W, Rymarenko O, Schünemann J, Dienemann C, Gunkel P, Mussil B, Krull J, Teichmann U, Groß U, Cordes VC, Dobbelstein M, Görlich D (2021). Neutralization of SARS-CoV-2 by highly potent, hyperthermostable, and mutation-tolerant nanobodies. EMBO J.

[12] Harmsen MM, De Haard HJ (2007). Properties, production, and applications of camelid single-domain antibody fragments. Appl Microbiol Biotechnol.

[13] Hassanzadeh-Ghassabeh G, Devoogdt N, De Pauw P, Vincke C, Muyldermans S (2013). Nanobodies and their potential applications. Nanomed.

[14] Hmila I, Saerens D, Ben Abderrazek R, Vincke C, Abidi N, Benlasfar Z, Govaert J, El Ayeb M, Bouhaouala-Zahar B, Muyldermans S (2010). A bispecific nanobody to provide full protection against lethal scorpion envenoming. FASEB J Off Publ Fed Am Soc Exp Biol.

[15] Igawa T, Tsunoda H, Kuramochi T, Sampei Z, Ishii S, Hattori K (2011). Engineering the variable region of therapeutic IgG antibodies. mAbs.

[16] Jacobs TM, Yumerefendi H, Kuhlman B, Leaver-Fay A (2015). SwiftLib: rapid degenerate-codon-library optimization through dynamic programming. Nucleic Acids Res.

[17] Jeliazkov JR, Frick R, Zhou J, Gray JJ (2021). Robustification of RosettaAntibody and Rosetta SnugDock. PLoS One.

[18] Jumper J, Evans R, Pritzel A, Green T, Figurnov M, Ronneberger O, Tunyasuvunakool K, Bates R, Žídek A, Potapenko A, Bridgland A, Meyer C, Kohl SAA, Ballard AJ, Cowie A, Romera-Paredes B, Nikolov S, Jain R, Adler J, Back T, Petersen S, Reiman D, Clancy E, Zielinski M, Steinegger M, Pacholska M, Berghammer T, Bodenstein S, Silver D, Vinyals O, Senior AW, Kavukcuoglu K, Kohli P, Hassabis D (2021). Highly accurate protein structure prediction with AlphaFold. Nature.

[19] Katti MV, Sami-Subbu R, Ranjekar PK, Gupta VS (2000). Amino acid repeat patterns in protein sequences: their diversity and structural-functional implications. Protein Sci Publ Protein Soc.

[20] Kessel AB-TN (2010) Introduction to proteins structure, function, and motion. Molecular Immunology. https://www.sciencedirect.com/journal/molecular-immunology.

[21] Klooster R, Maassen BTH, Stam JC, Hermans PW, Ten Haaft MR, Detmers FJM, de Haard HJ, Post JA, Theo Verrips C (2007). Improved anti-IgG and HSA affinity ligands: clinical application of VHH antibody technology. J Immunol Methods.

[22] Korotkov KV, Pardon E, Steyaert J (1993). Hol WGJ (2009) Crystal structure of the N-terminal domain of the secretin GspD from ETEC determined with the assistance of a nanobody. Struct Lond Engl.

[23] Kumar S, Robins R, Buck P, Hickling T, Thangakani A, Li L, Singh S, Gromiha M (2015). Biopharmaceutical informatics: applications of computation in biologic drug discovery and development.

[24] Li F, Vijayasankaran N, Shen AY, Kiss R, Amanullah A (2010). Cell culture processes for monoclonal antibody production. mAbs.

[25] Lipinski CA, Lombardo F, Dominy BW, Feeney PJ (2001). Experimental and computational approaches to estimate solubility and permeability in drug discovery and development settings. Adv Drug Deliv Rev.

[26] Luo H, Nijveen H (2014). Understanding and identifying amino acid repeats. Brief Bioinform.

[27] Muyldermans S, Atarhouch T, Saldanha J, Barbosa JA, Hamers R (1994). Sequence and structure of VH domain from naturally occurring camel heavy chain immunoglobulins lacking light chains. Protein Eng.

[28] Nakazawa M, Mukumoto M, Miyatake K (2010). Production and purification of monoclonal antibodies. Methods Mol Biol Clifton NJ.

[29] Nguyen-Duc T, Peeters E, Muyldermans S, Charlier D, Hassanzadeh-Ghassabeh G (2013). Nanobody®-based chromatin immunoprecipitation/micro-array analysis for genome-wide identification of transcription factor DNA binding sites. Nucleic Acids Res.

[30] Pardon E, Laeremans T, Triest S, Rasmussen SGF, Wohlkönig A, Ruf A, Muyldermans S, Hol WGJ, Kobilka BK, Steyaert J (2014). A general protocol for the generation of nanobodies for structural biology. Nat Protoc.

[31] Perchiacca JM, Ladiwala ARA, Bhattacharya M, Tessier PM (2012). Aggregation-resistant domain antibodies engineered with charged mutations near the edges of the complementarity-determining regions. Protein Eng Des Sel PEDS.

[32] Perchiacca JM, Lee CC, Tessier PM (2014). Optimal charged mutations in the complementarity-determining regions that prevent domain antibody aggregation are dependent on the antibody scaffold. Protein Eng Des Sel PEDS.

[33] Phatak SS, Stephan CC, Cavasotto CN (2009). High-throughput and in silico screenings in drug discovery. Expert Opin Drug Discovery.

[34] Rock EP, Sibbald PR, Davis MM, Chien YH (1994). CDR3 length in antigen-specific immune receptors. J Exp Med.

[35] Rothbauer U, Zolghadr K, Tillib S, Nowak D, Schermelleh L, Gahl A, Backmann N, Conrath K, Muyldermans S, Cardoso MC, Leonhardt H (2006). Targeting and tracing antigens in live cells with fluorescent nanobodies. Nat Methods.

[36] Sela-Culang I, Kunik V, Ofran Y (2013). The structural basis of antibody-antigen recognition. Front Immunol.

[37] Sevy AM, Chen M-T, Castor M, Sylvia T, Krishnamurthy H, Ishchenko A, Hsieh C-M (2020). Structure- and sequence-based design of synthetic single-domain antibody libraries. Protein Eng Des Sel.

[38] Stefan MA, Light YK, Schwedler JL, McIlroy PR, Courtney CM, Saada EA, Thatcher CE, Phillips AM, Bourguet FA, Mageeney CM, McCloy SA, Collette NM, Negrete OA, Schoeniger JS, Weilhammer DR, Harmon B (2021). Development of potent and effective synthetic SARS-CoV-2 neutralizing nanobodies. mAbs.

[39] Tomita M, Tsumoto K (2011). Hybridoma technologies for antibody production. Immunotherapy.

[40] Vaneycken I, Devoogdt N, Van Gassen N, Vincke C, Xavier C, Wernery U, Muyldermans S, Lahoutte T, Caveliers V (2011). Preclinical screening of anti-HER2 nanobodies for molecular imaging of breast cancer. FASEB J Off Publ Fed Am Soc Exp Biol.

[41] Voss JE (2021). Engineered single-domain antibodies tackle COVID variants. Nature.

[42] Wagner HJ, Wehrle S, Weiss E, Cavallari M, Weber W (2018). A two-step approach for the design and generation of nanobodies. Int J Mol Sci.

[43] Wilson S, Howell S (2002). High-throughput screening in the diagnostics industry. Biochem Soc Trans.

[44] Wu Y, Li C, Xia S, Tian X, Kong Y, Wang Z, Gu C, Zhang R, Tu C, Xie Y, Yang Z, Lu L, Jiang S, Ying T (2020). Identification of human single-domain antibodies against SARS-CoV-2. Cell Host Microbe.

[45] Xu J, Xu K, Jung S, Conte A, Lieberman J, Muecksch F, Lorenzi JCC, Park S, Schmidt F, Wang Z, Huang Y, Luo Y, Nair MS, Wang P, Schulz JE, Tessarollo L, Bylund T, Chuang G-Y, Olia AS, Stephens T, Teng I-T, Tsybovsky Y, Zhou T, Munster V, Ho DD, Hatziioannou T, Bieniasz PD, Nussenzweig MC, Kwong PD, Casellas R (2021). Nanobodies from camelid mice and llamas neutralize SARS-CoV-2 variants. Nature.

[46] Yavuz S, Komsuoğlu Çelikyurt FI (2021) Antiviral treatment of COVID-19: an update. Turk. J Med Sci 10.3906/sag-2106-250 PMC8771049 34391321

[47] Zare H, Aghamollaei H, Hosseindokht M, Heiat M, Razei A, Bakherad H (2021). Nanobodies, the potent agents to detect and treat the coronavirus infections: a systematic review. Mol Cell Probes.

[48] Zemlin M, Klinger M, Link J, Zemlin C, Bauer K, Engler JA, Schroeder HW, Kirkham PM (2003). Expressed murine and human CDR-H3 intervals of equal length exhibit distinct repertoires that differ in their amino acid composition and predicted range of structures. J Mol Biol.

[49] Zupancic JM, Desai AA, Schardt JS, Pornnoppadol G, Makowski EK, Smith MD, Kennedy AA, de Mattos G, Barbosa M, Cascalho M, Lanigan TM, Tai AW, Tessier PM (2021). Directed evolution of potent neutralizing nanobodies against SARS-CoV-2 using CDR-swapping mutagenesis. Cell Chem Biol.

[50] Zupancic JM, Schardt JS, Desai AA, Makowski EK, Smith MD, Pornnoppadol G, de Mattos G, Barbosa M, Cascalho M, Lanigan TM, Tessier PM (2021). Engineered multivalent nanobodies potently and broadly neutralize SARS-CoV-2 variants. Adv Ther.

