# Supplementary material for: Studying the characteristics of nanobody CDR regions based on sequence analysis in combination with 3D structures
Source: J Genet Eng Biotechnol. 2022 Nov 21;20:157. doi: 10.1186/s43141-022-00439-9 (PMC9684374; doi:10.1186/s43141-022-00439-9)
Supplement: Supplementary file 1 — Additional file 1: Figure S1. For the CDR2 loops, most of the residues showed strong correlation with R2 > 0.8 (except for G and P). Figure S2. Correlation between the highest occurrence of amino acids and CDR3 lengths. Figure S3. Occurrence of repeat motifs formed by corresponding amino acids in each CDR region. (A) poly amino acid repeats, (B) oligo repeats. [file 43141_2022_439_MOESM1_ESM.pdf]

Supplement Figure 1

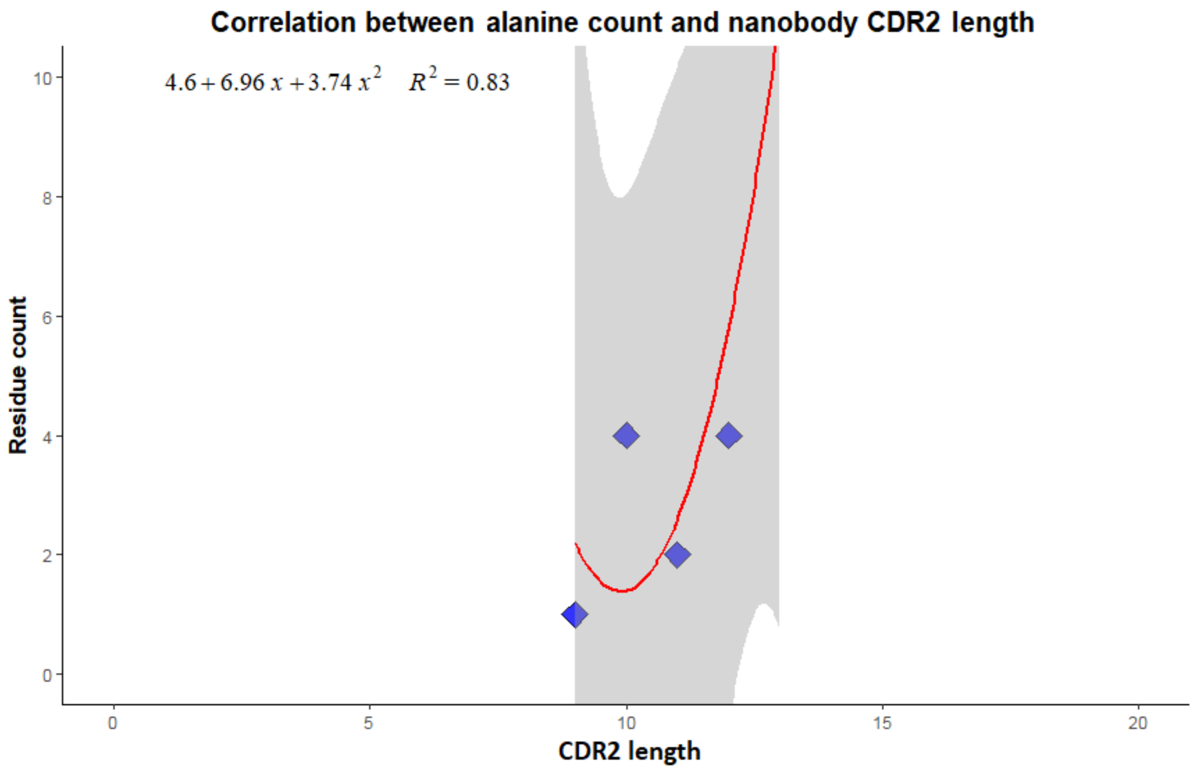

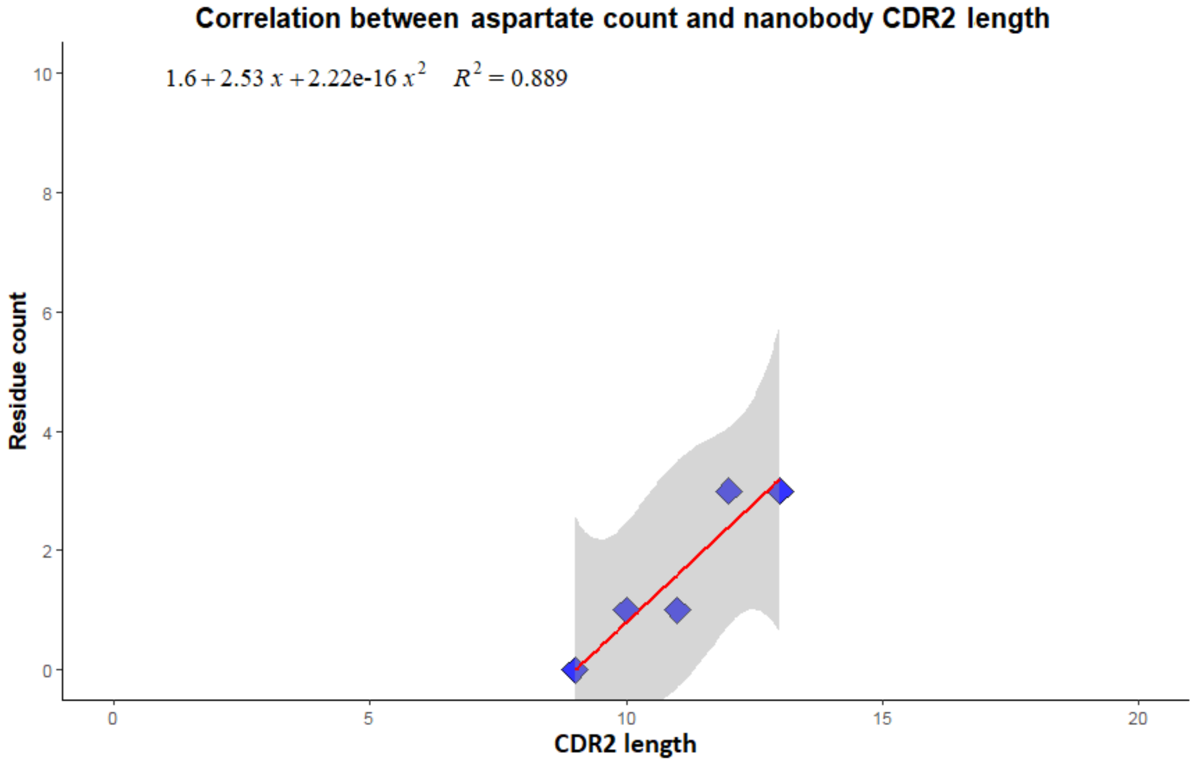

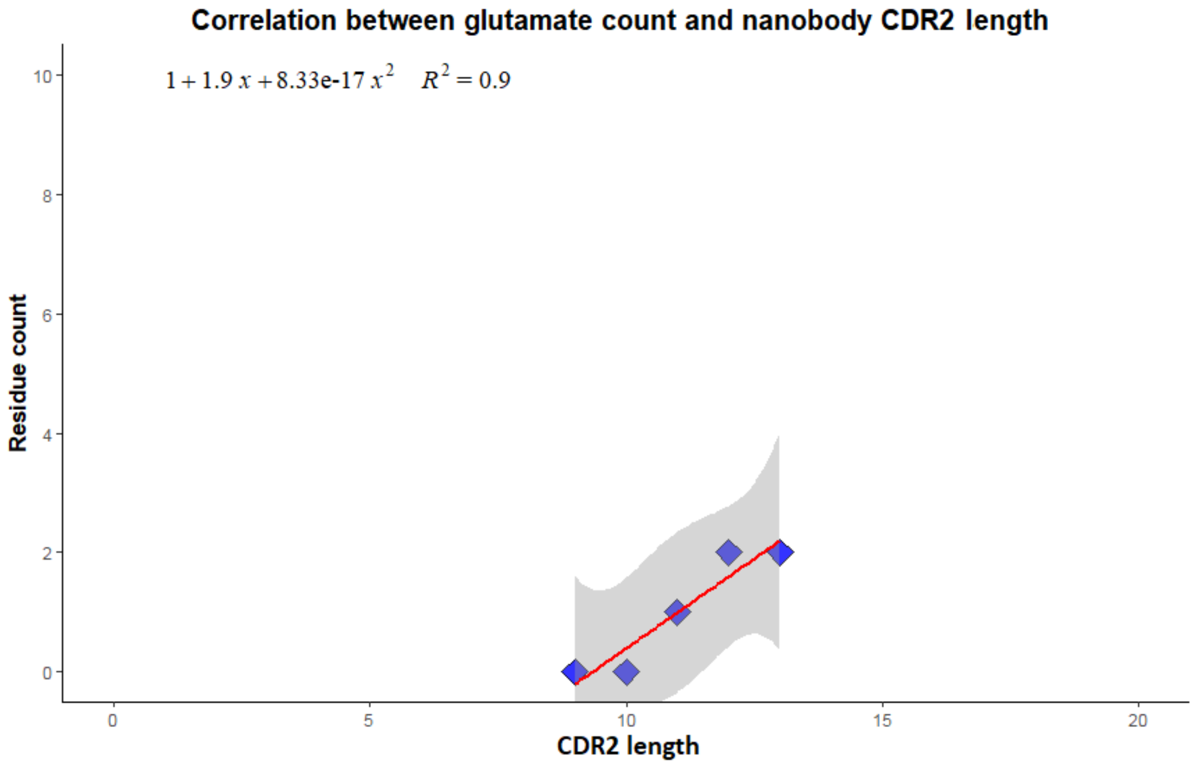
Correlation between glutamate count and nanobody CDR2 length
$1 + 1.9\,x + 8.33\text{e-}17\,x^2 \quad R^2 = 0.9$
Residue count
CDR2 length
0
2
4
6
8
10
0
5
10
15
20

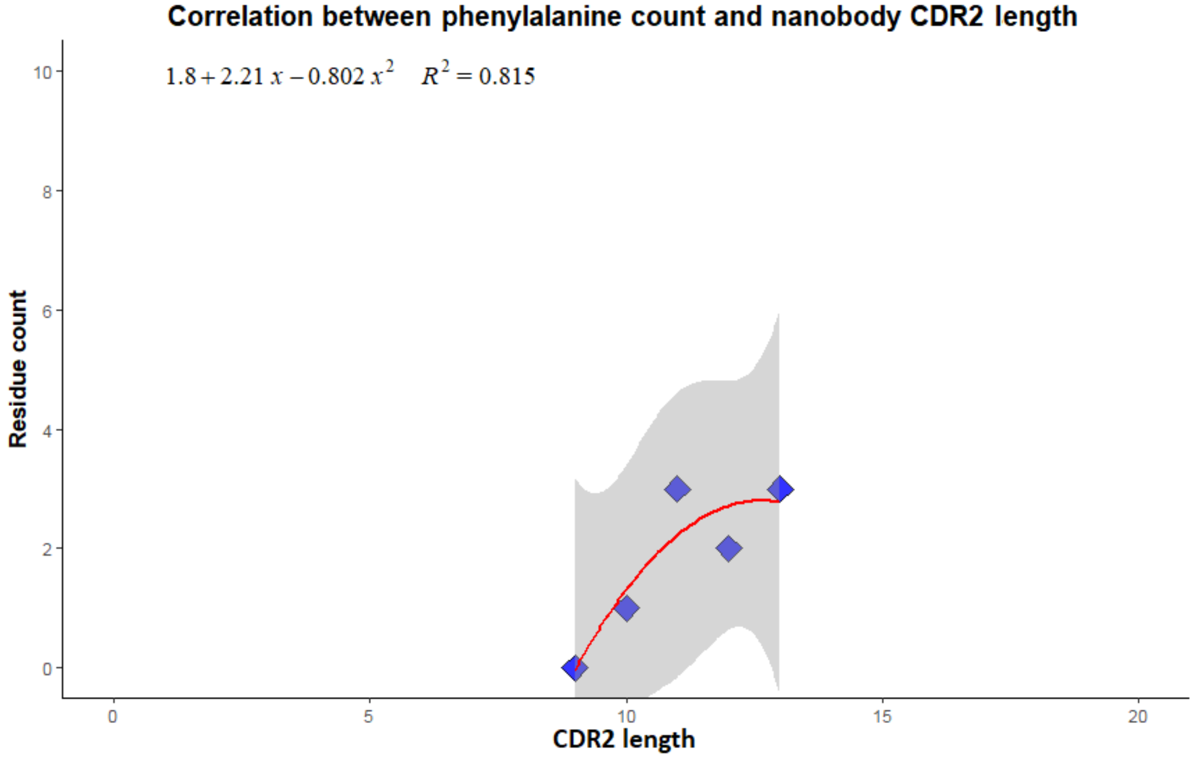
Correlation between phenylalanine count and nanobody CDR2 length
$1.8 + 2.21\,x - 0.802\,x^2 \quad R^2 = 0.815$
Residue count
CDR2 length
0
2
4
6
8
10
0
5
10
15
20

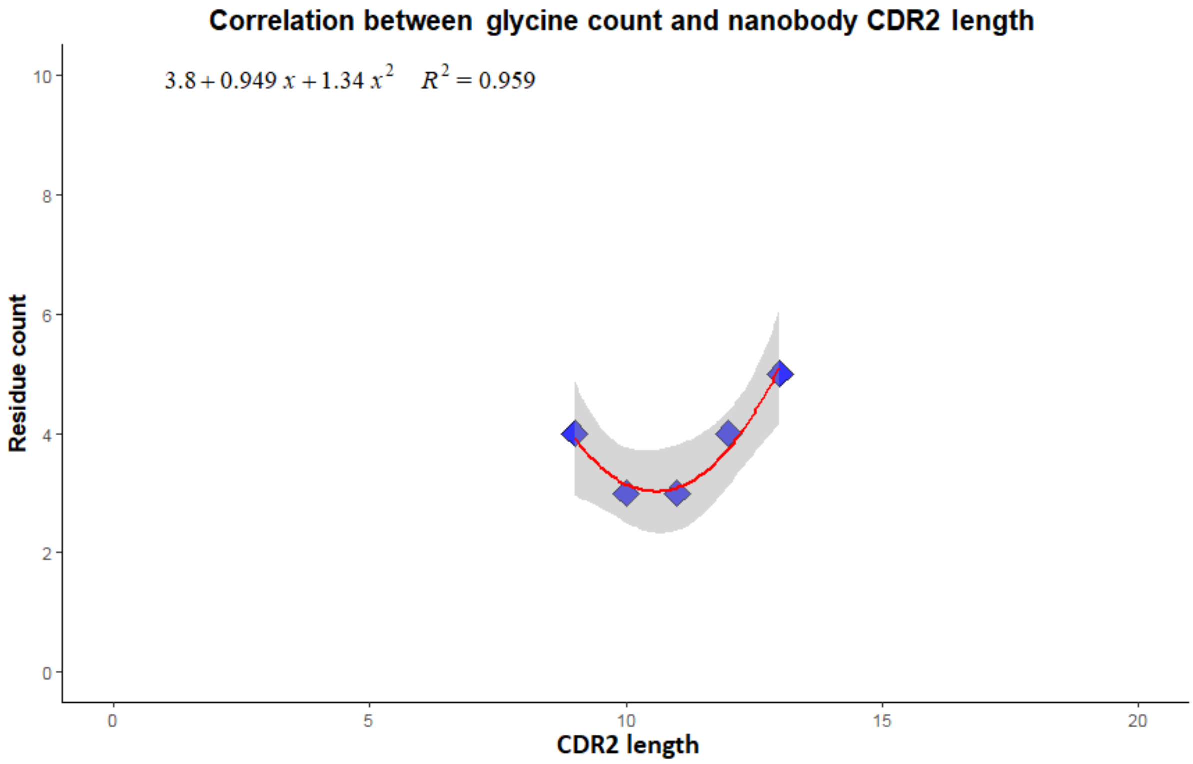
Correlation between glycine count and nanobody CDR2 length
3.8 + 0.949 x + 1.34 x2   R2 = 0.959
Residue count
CDR2 length
0
2
4
6
8
10
0
5
10
15
20

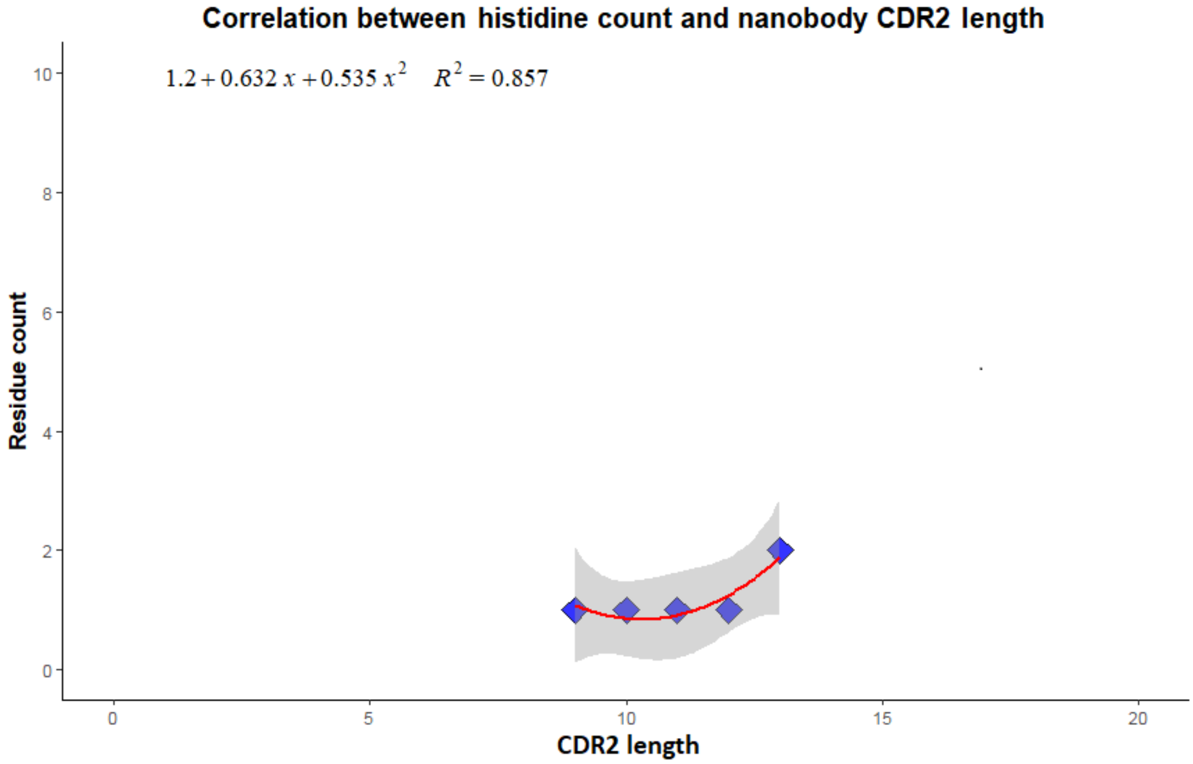
Correlation between histidine count and nanobody CDR2 length
1.2 + 0.632 x + 0.535 x2   R2 = 0.857
Residue count
CDR2 length
0
2
4
6
8
10
0
5
10
15
20

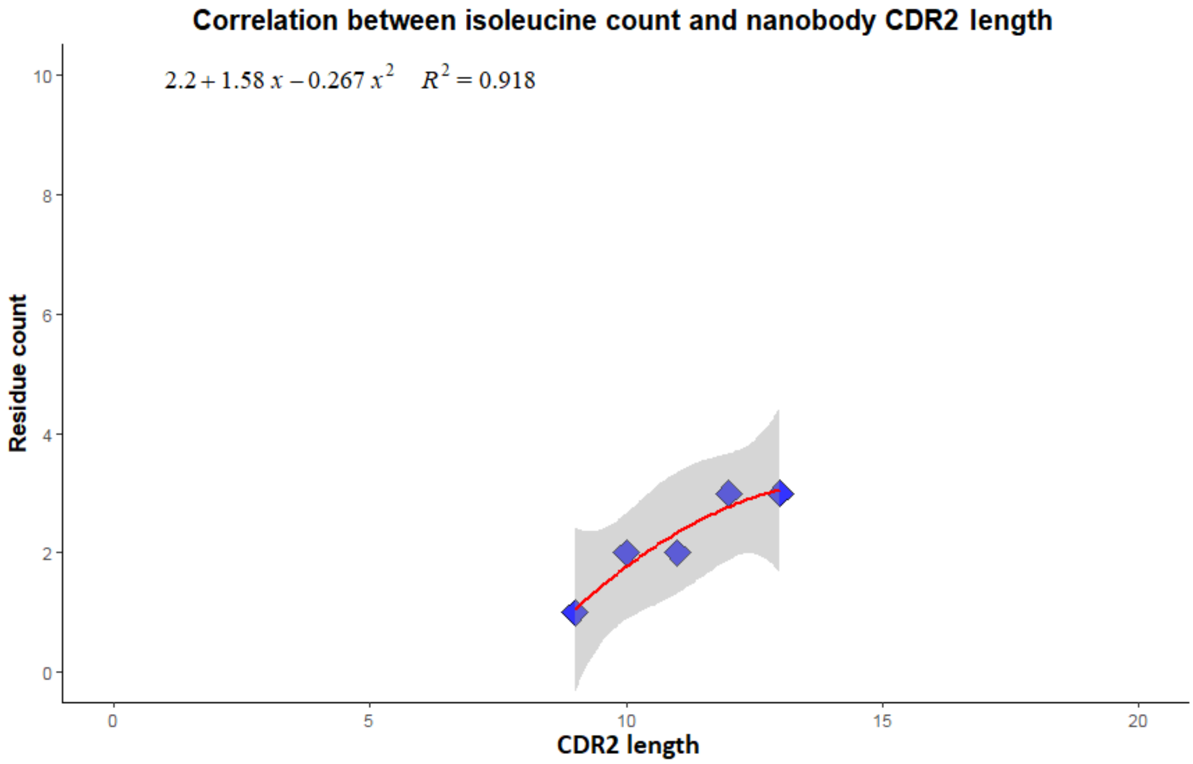

Correlation between isoleucine count and nanobody CDR2 length
$2.2 + 1.58\,x - 0.267\,x^2$ $R^2 = 0.918$
Residue count
0
2
4
6
8
10
0
5
10
15
20
CDR2 length

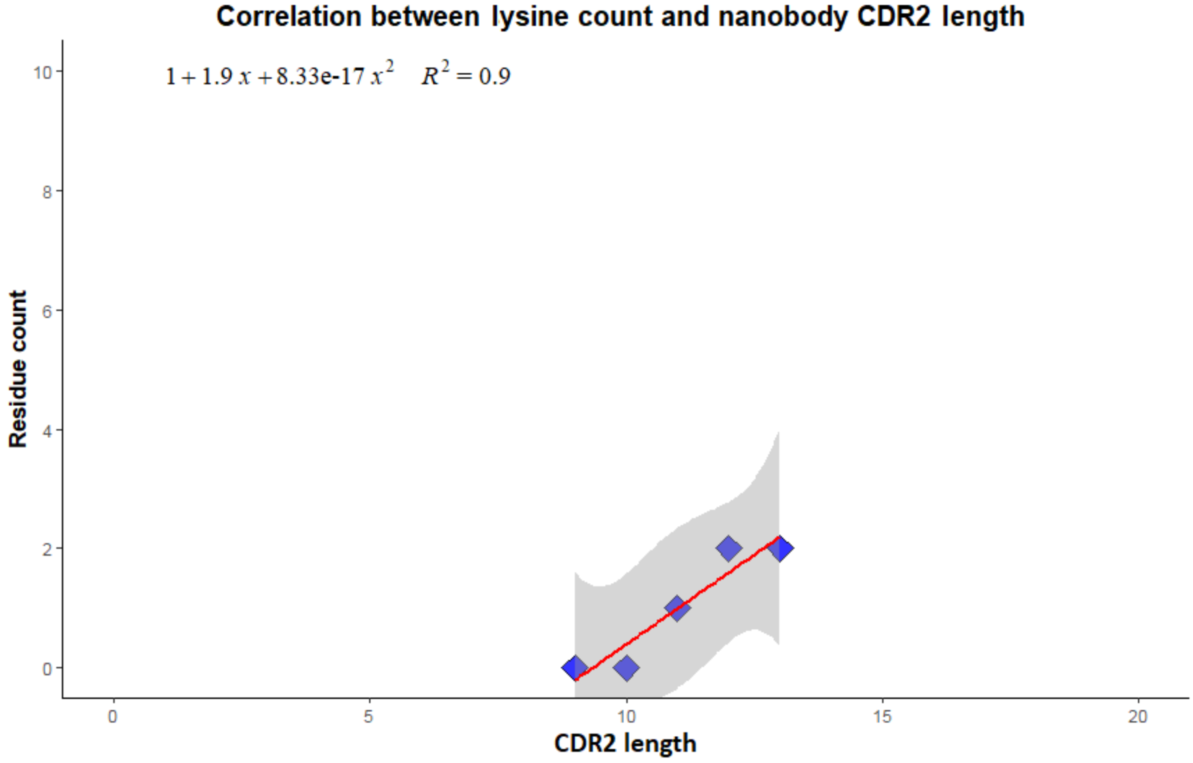

Correlation between lysine count and nanobody CDR2 length
$1 + 1.9\,x + 8.33\text{e-}17\,x^2$ $R^2 = 0.9$
Residue count
0
2
4
6
8
10
0
5
10
15
20
CDR2 length

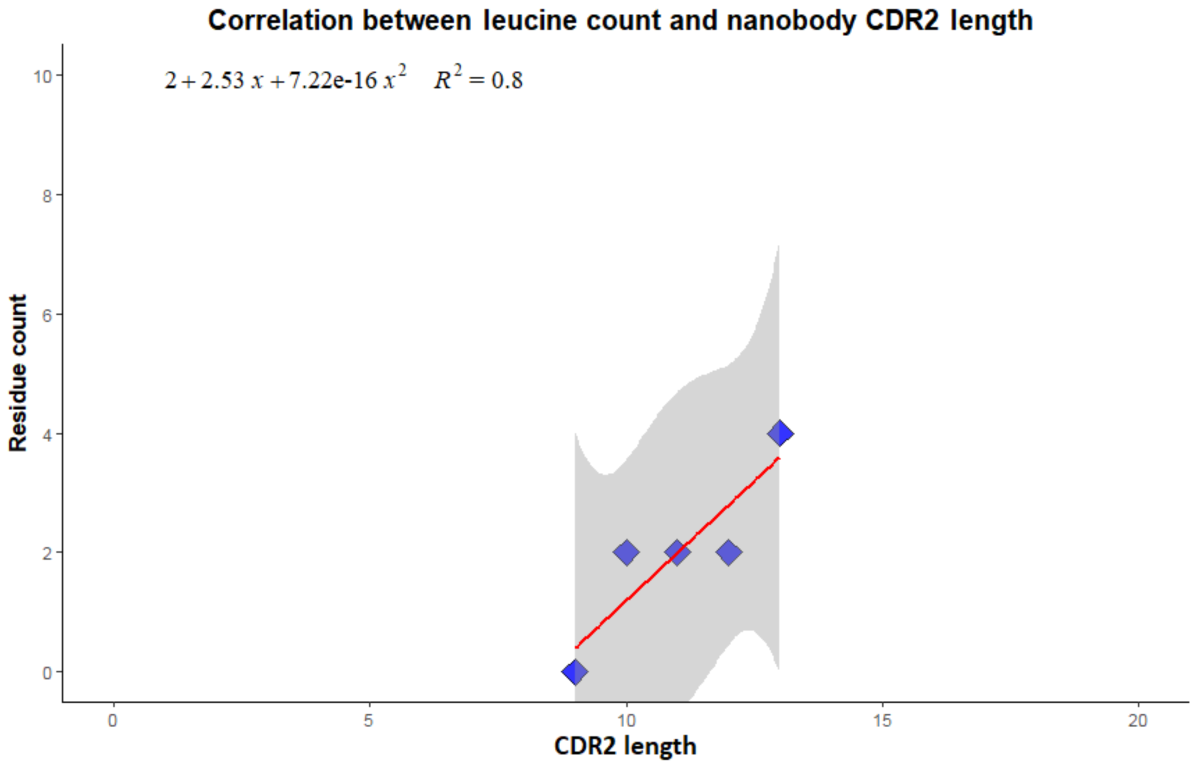
Correlation between leucine count and nanobody CDR2 length
2 + 2.53 x + 7.22e-16 x^2 R^2 = 0.8
Residue count
CDR2 length
0
2
4
6
8
10
0
5
10
15
20

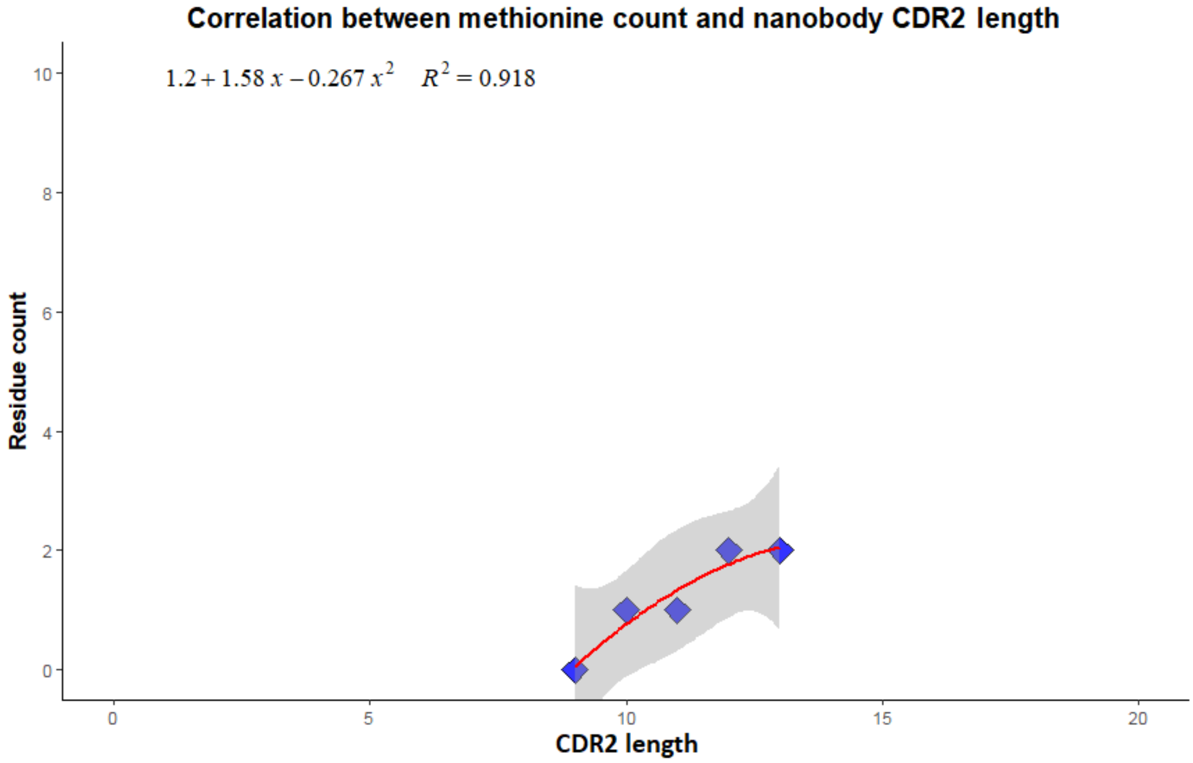
Correlation between methionine count and nanobody CDR2 length
1.2 + 1.58 x − 0.267 x^2 R^2 = 0.918
Residue count
CDR2 length
0
2
4
6
8
10
0
5
10
15
20

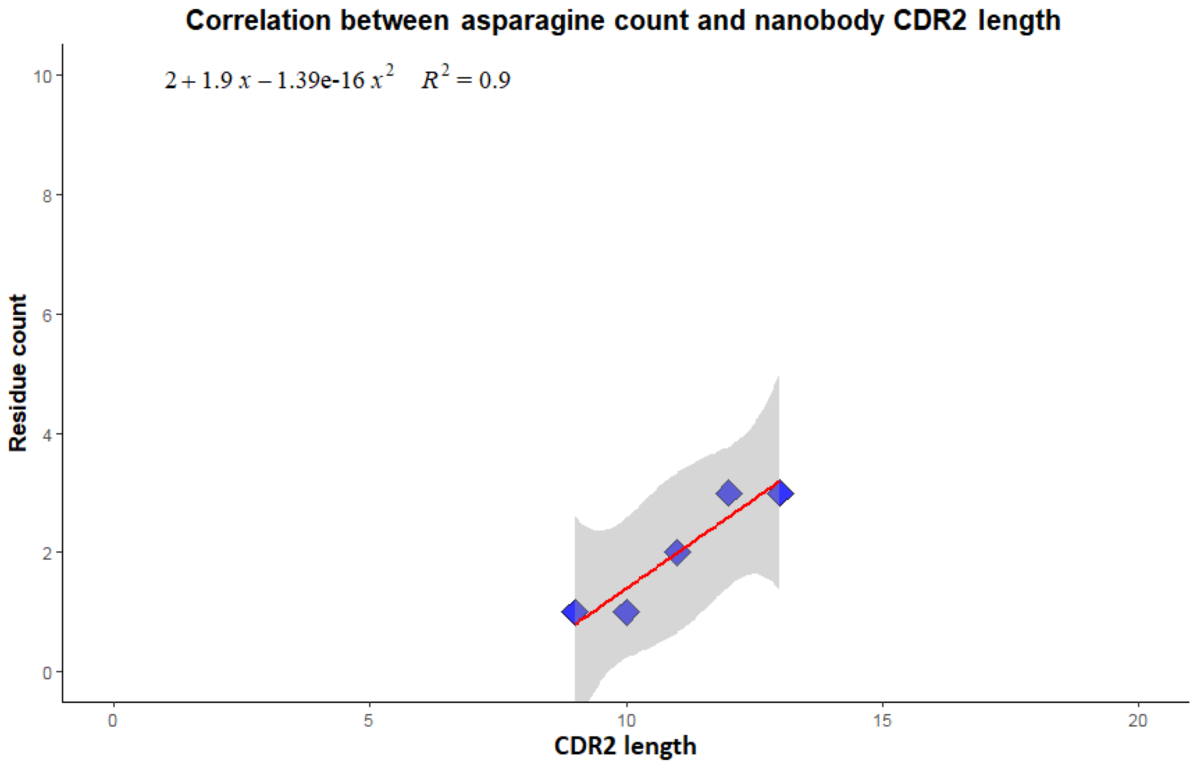
Correlation between asparagine count and nanobody CDR2 length
$2 + 1.9\,x - 1.39\text{e-}16\,x^2 \quad R^2 = 0.9$
Residue count
0
2
4
6
8
10
0
5
10
15
20
CDR2 length

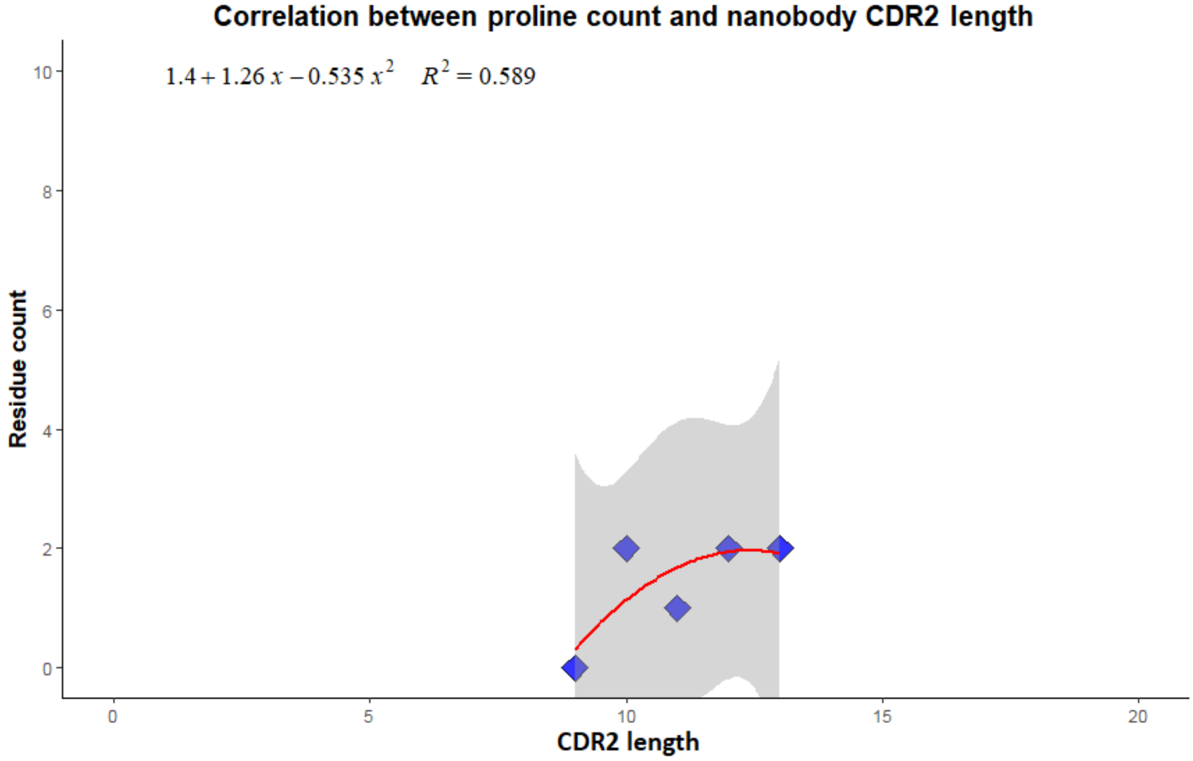
Correlation between proline count and nanobody CDR2 length
$1.4 + 1.26\,x - 0.535\,x^2 \quad R^2 = 0.589$
Residue count
0
2
4
6
8
10
0
5
10
15
20
CDR2 length

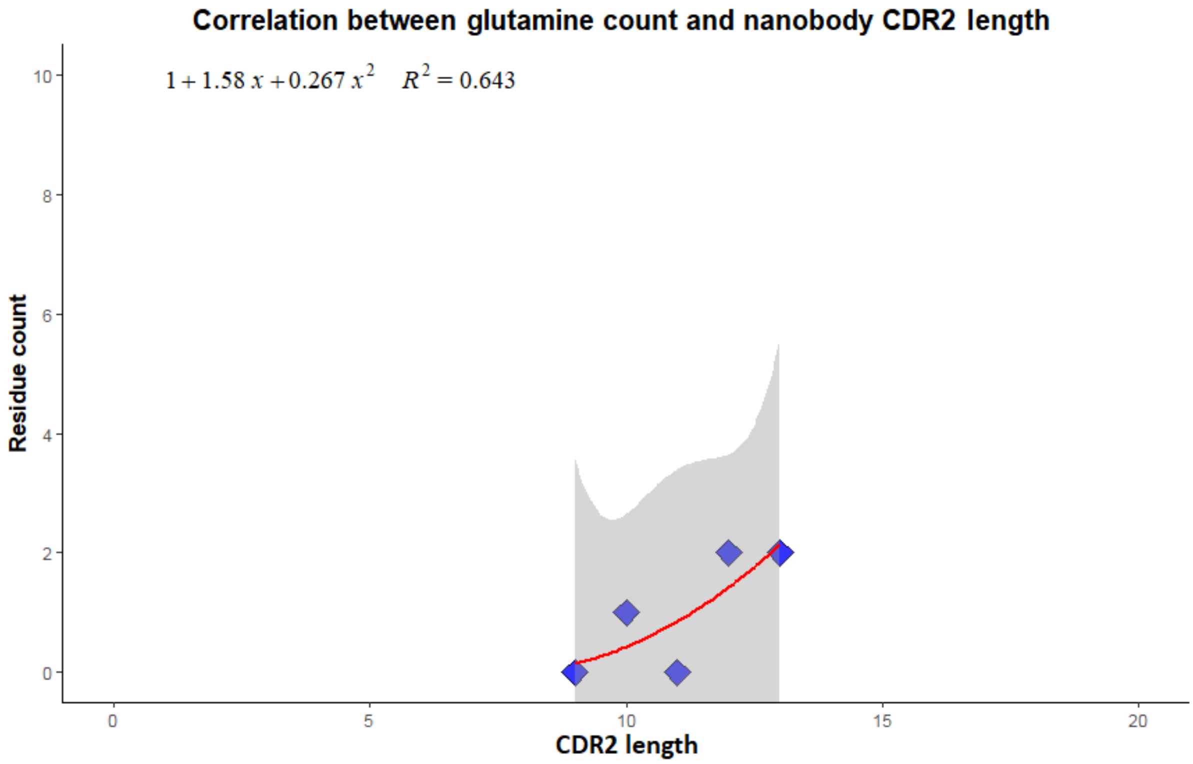

Correlation between glutamine count and nanobody CDR2 length
$1 + 1.58\,x + 0.267\,x^2 \quad R^2 = 0.643$
Residue count
CDR2 length
0
2
4
6
8
10
0
5
10
15
20

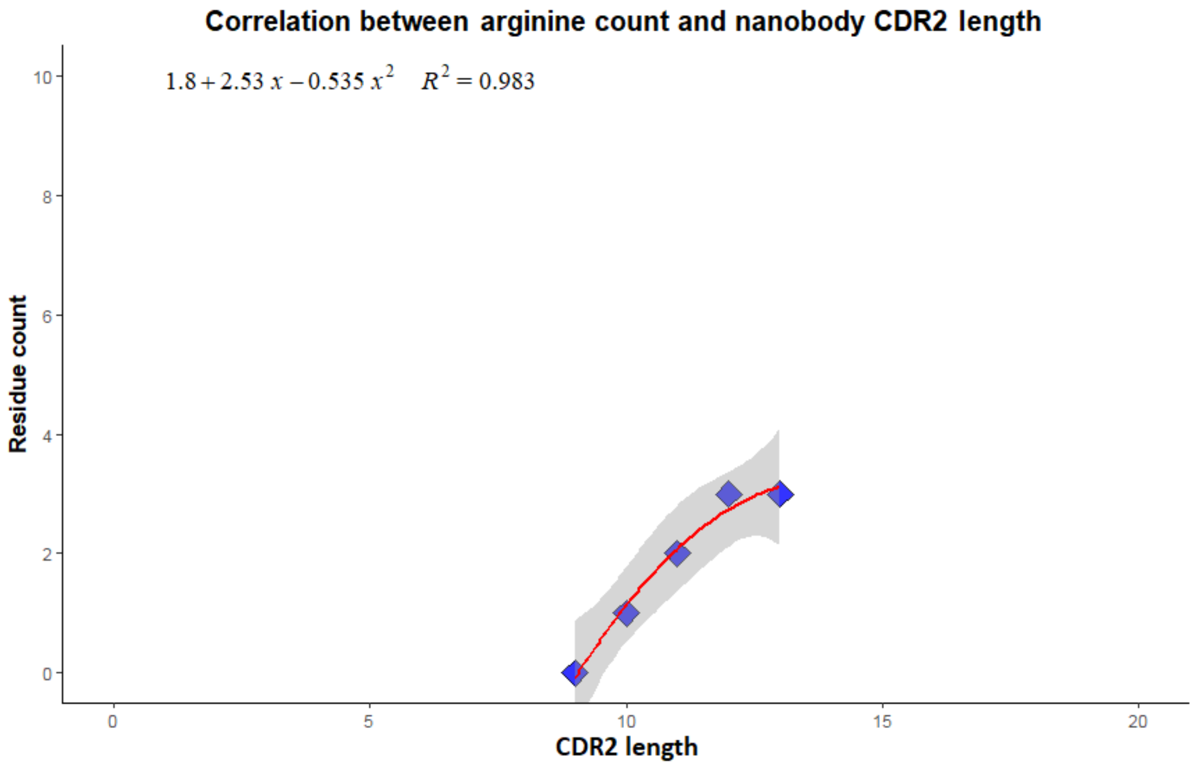

Correlation between arginine count and nanobody CDR2 length
$1.8 + 2.53\,x - 0.535\,x^2 \quad R^2 = 0.983$
Residue count
CDR2 length
0
2
4
6
8
10
0
5
10
15
20

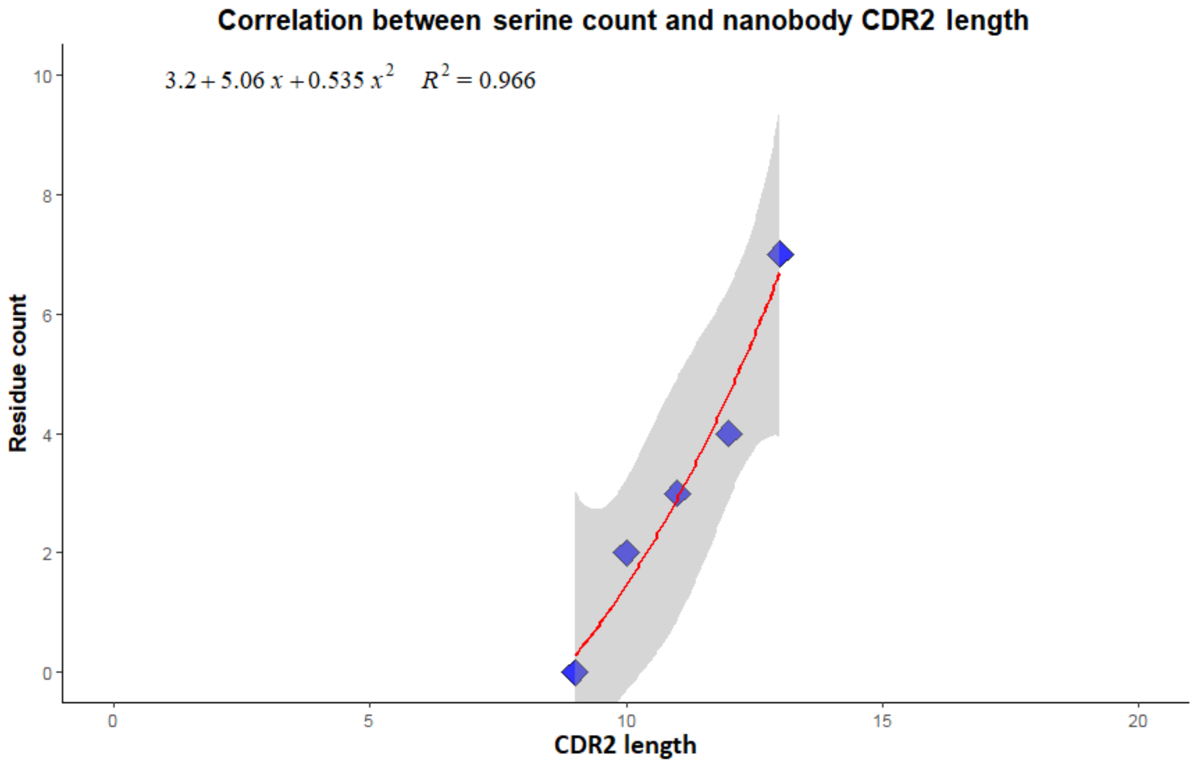
Correlation between serine count and nanobody CDR2 length
3.2 + 5.06 x + 0.535 x^2   R^2 = 0.966
Residue count
CDR2 length
0
2
4
6
8
10
0
5
10
15
20

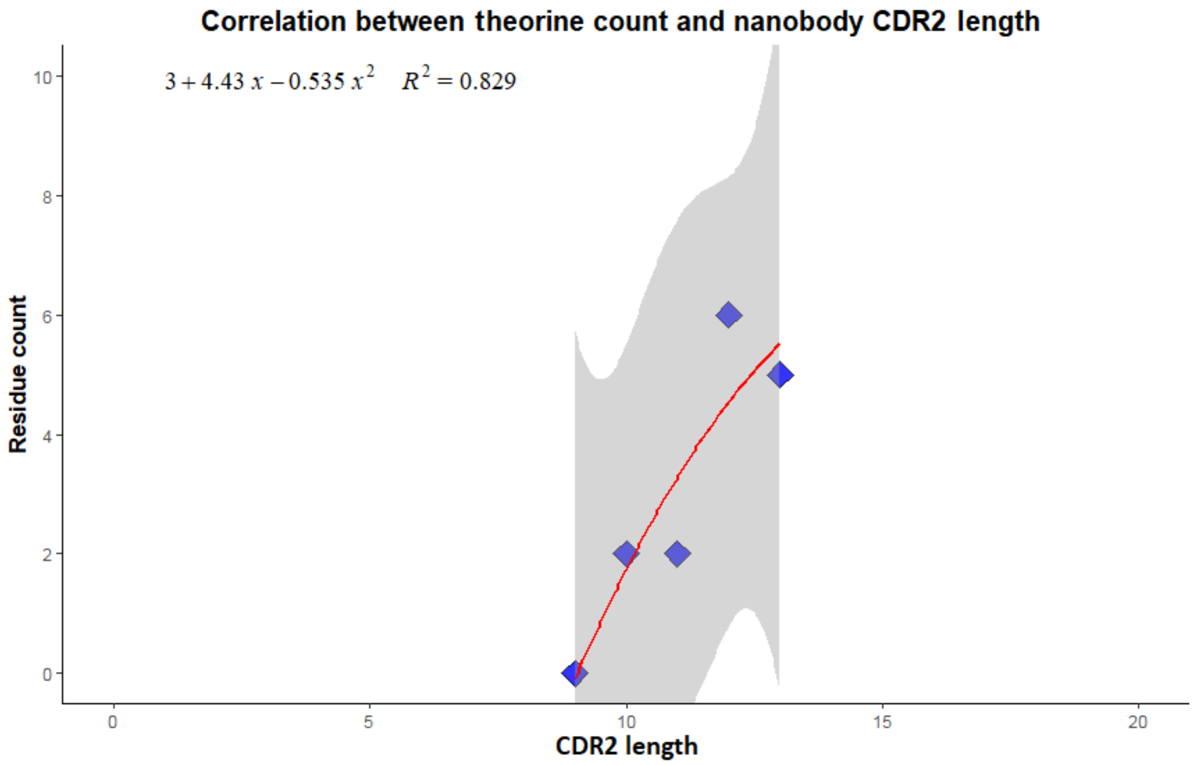
Correlation between theorine count and nanobody CDR2 length
3 + 4.43 x − 0.535 x^2   R^2 = 0.829
Residue count
CDR2 length
0
2
4
6
8
10
0
5
10
15
20

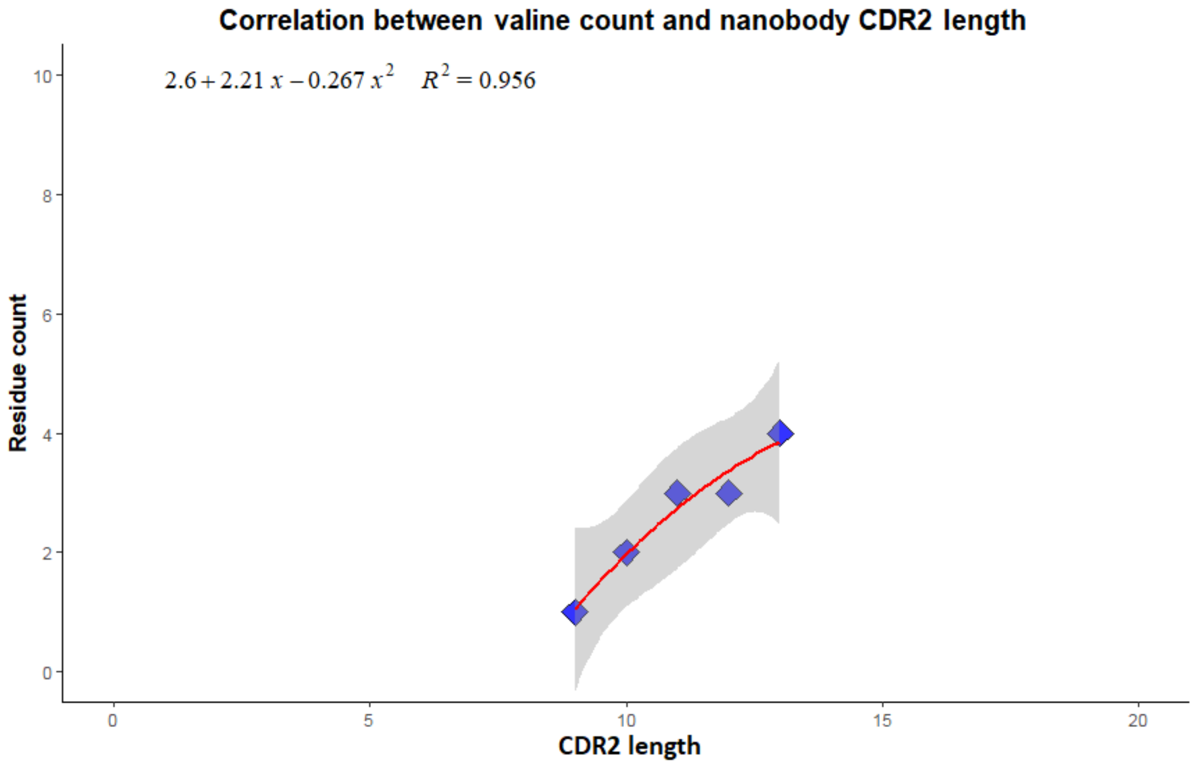
Correlation between valine count and nanobody CDR2 length
2.6 + 2.21 x − 0.267 x^2   R^2 = 0.956
Residue count
CDR2 length
0
2
4
6
8
10
0
5
10
15
20

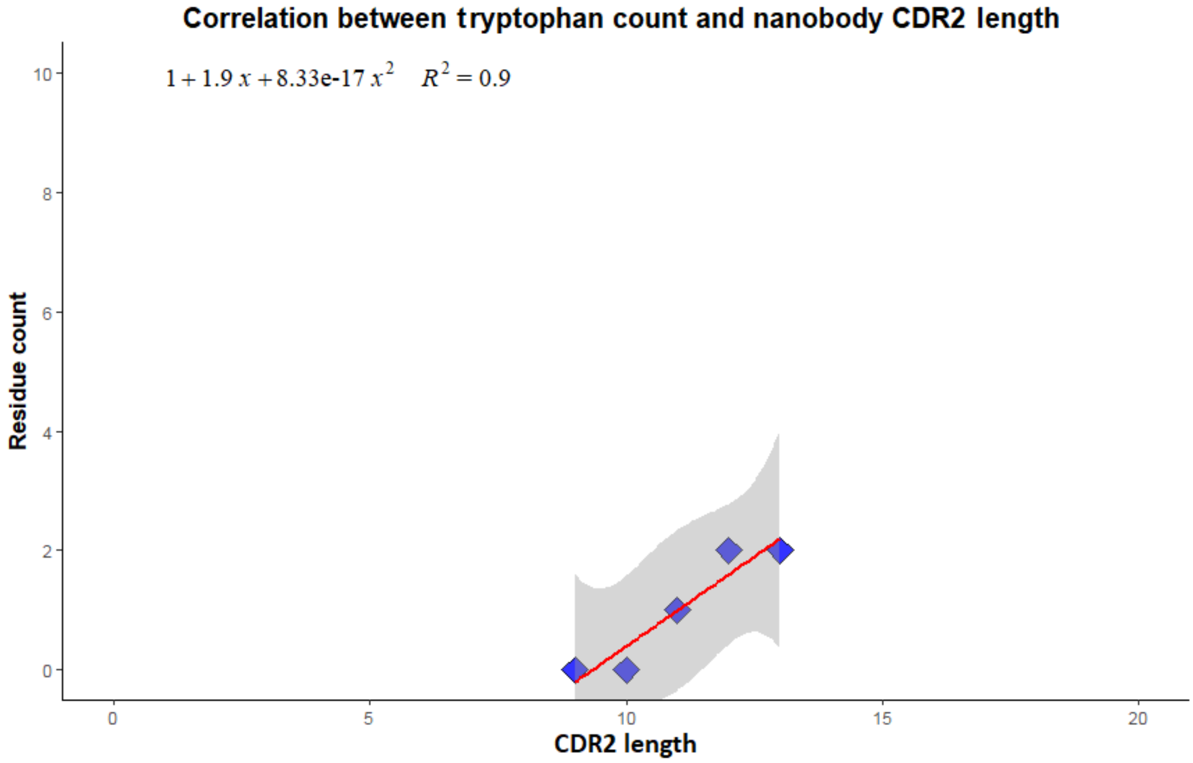
Correlation between tryptophan count and nanobody CDR2 length
1 + 1.9 x + 8.33e-17 x^2   R^2 = 0.9
Residue count
CDR2 length
0
2
4
6
8
10
0
5
10
15
20

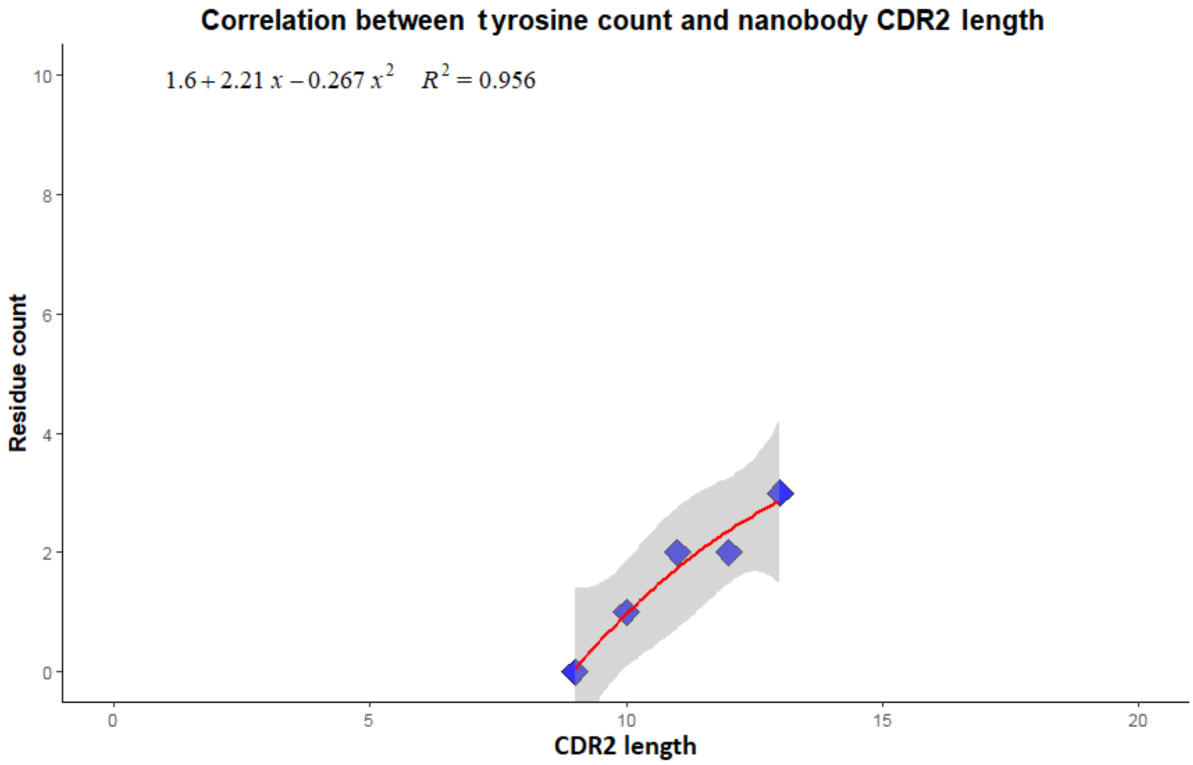

Supplement Figure 1. Correlation between the highest occurrence of amino acids and CDR2 lengths.

Supplement Figure 2

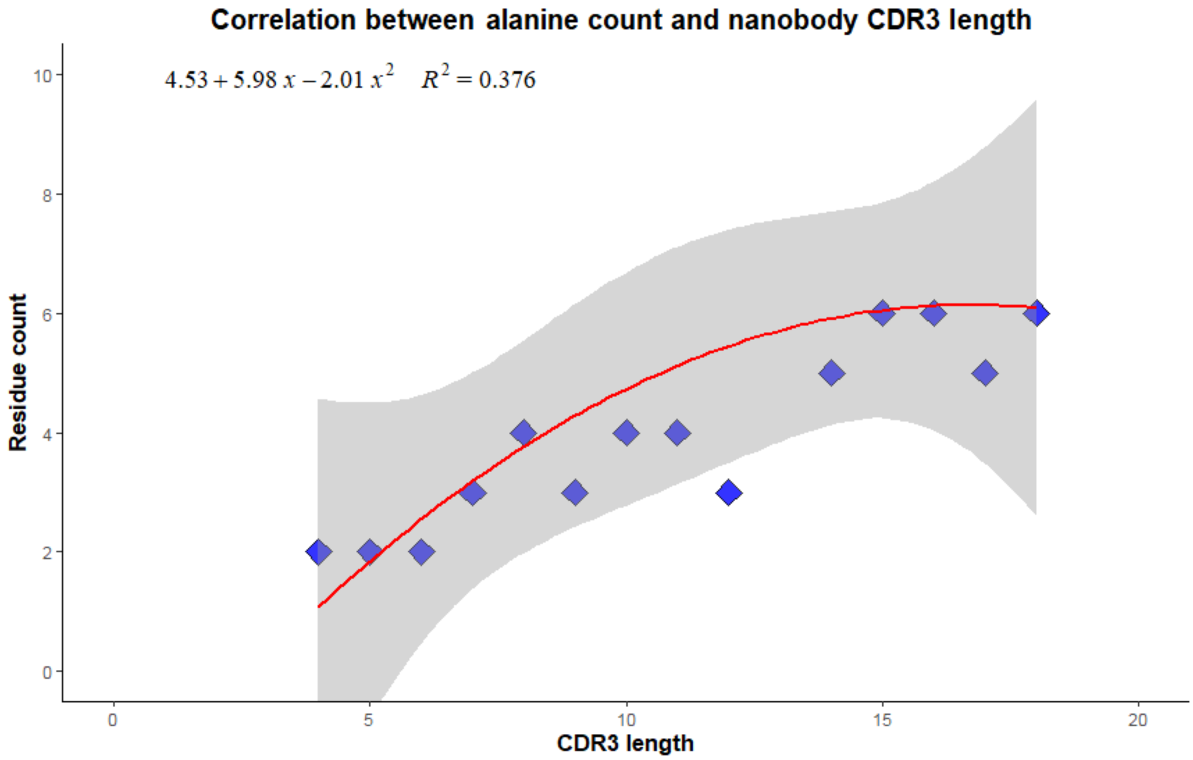

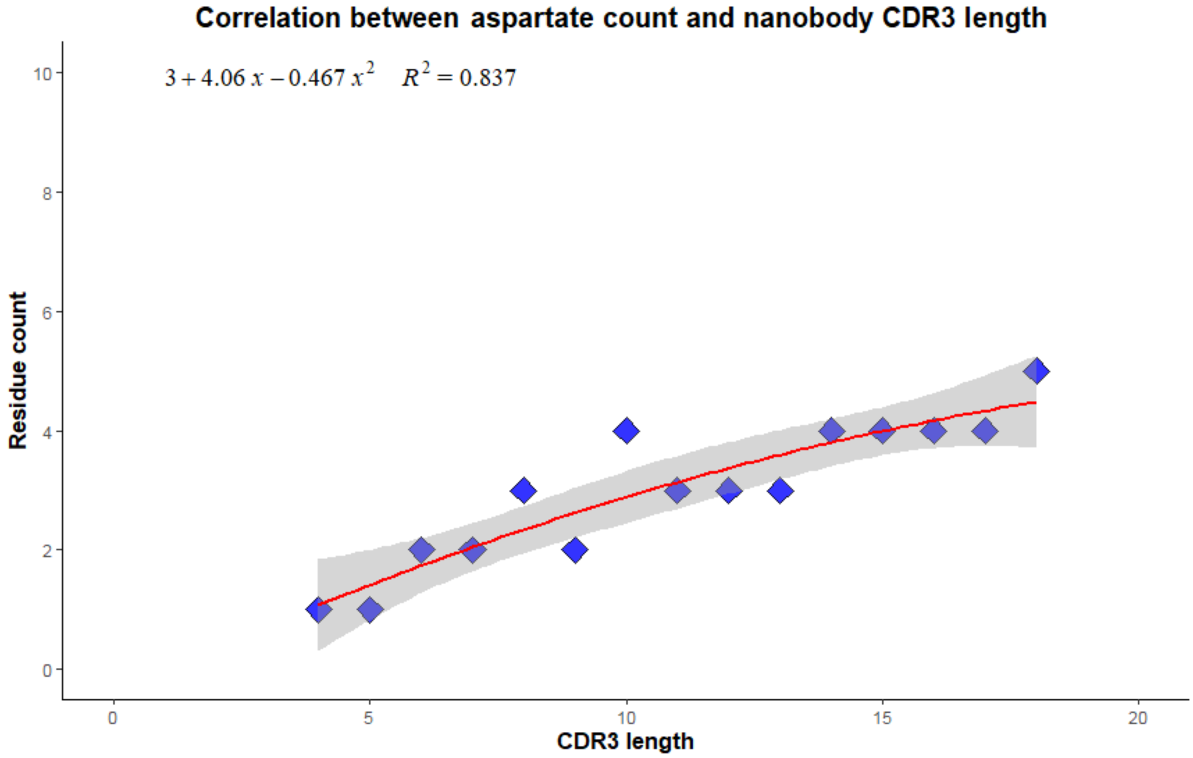

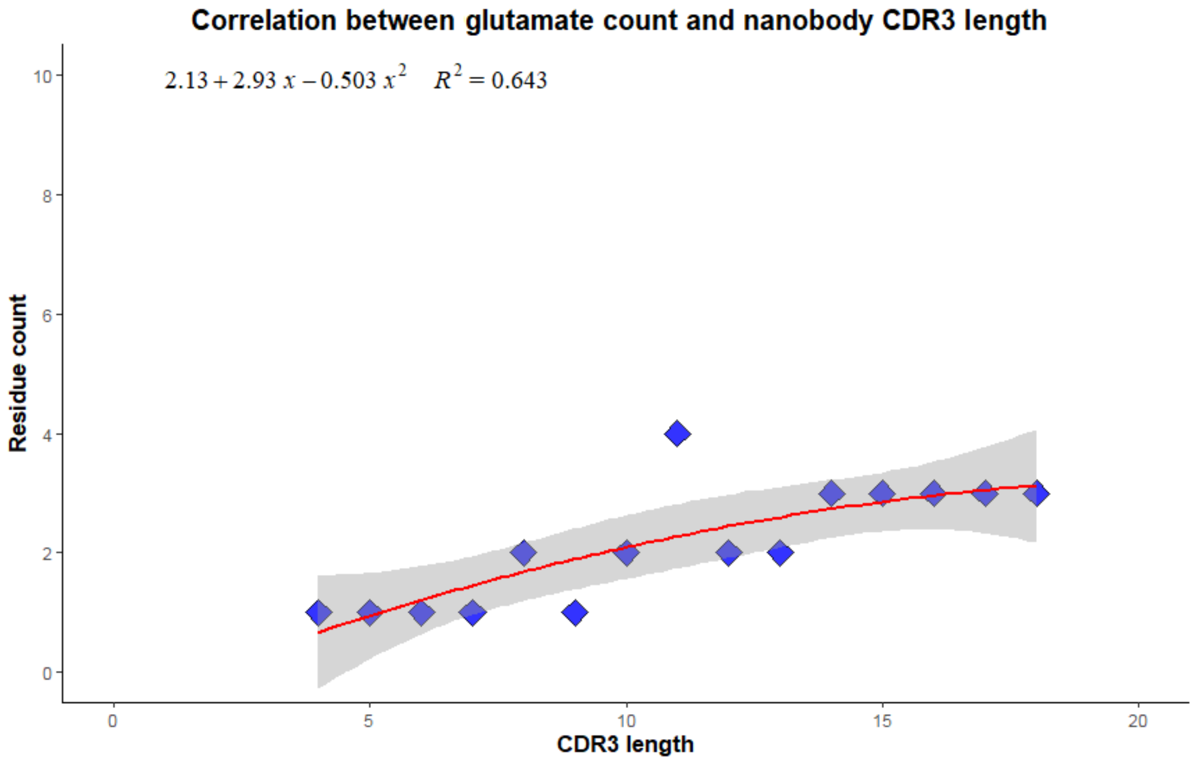
Correlation between glutamate count and nanobody CDR3 length
$2.13 + 2.93\,x - 0.503\,x^2 \quad R^2 = 0.643$
Residue count
CDR3 length
0
2
4
6
8
10
0
5
10
15
20

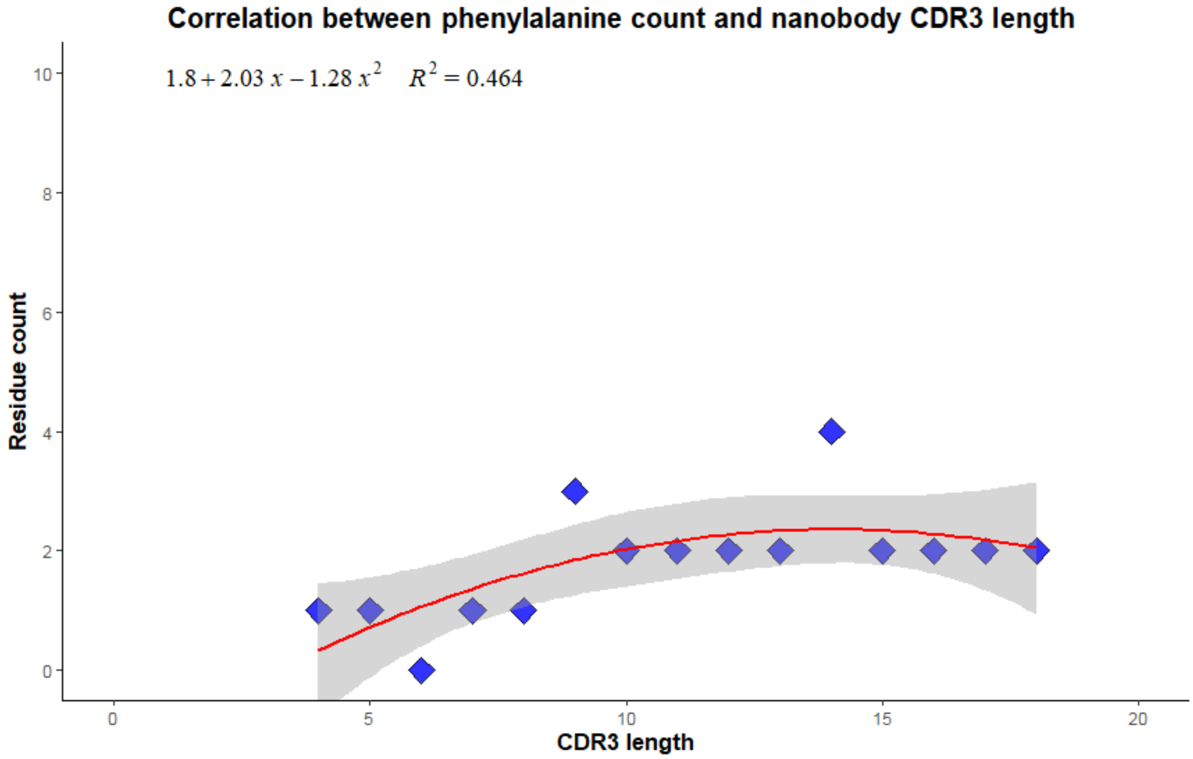
Correlation between phenylalanine count and nanobody CDR3 length
$1.8 + 2.03\,x - 1.28\,x^2 \quad R^2 = 0.464$
Residue count
CDR3 length
0
2
4
6
8
10
0
5
10
15
20

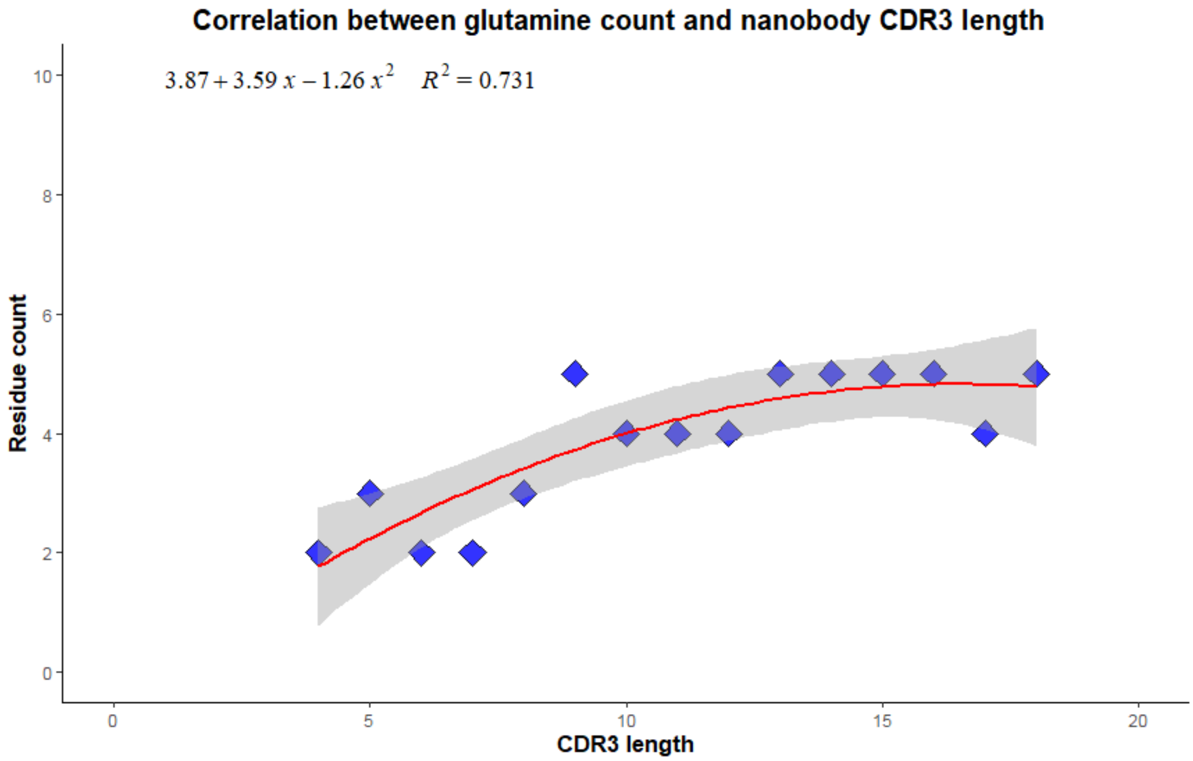

Correlation between glutamine count and nanobody CDR3 length
$3.87 + 3.59\,x - 1.26\,x^2 \quad R^2 = 0.731$
Residue count
CDR3 length
0
2
4
6
8
10
0
5
10
15
20

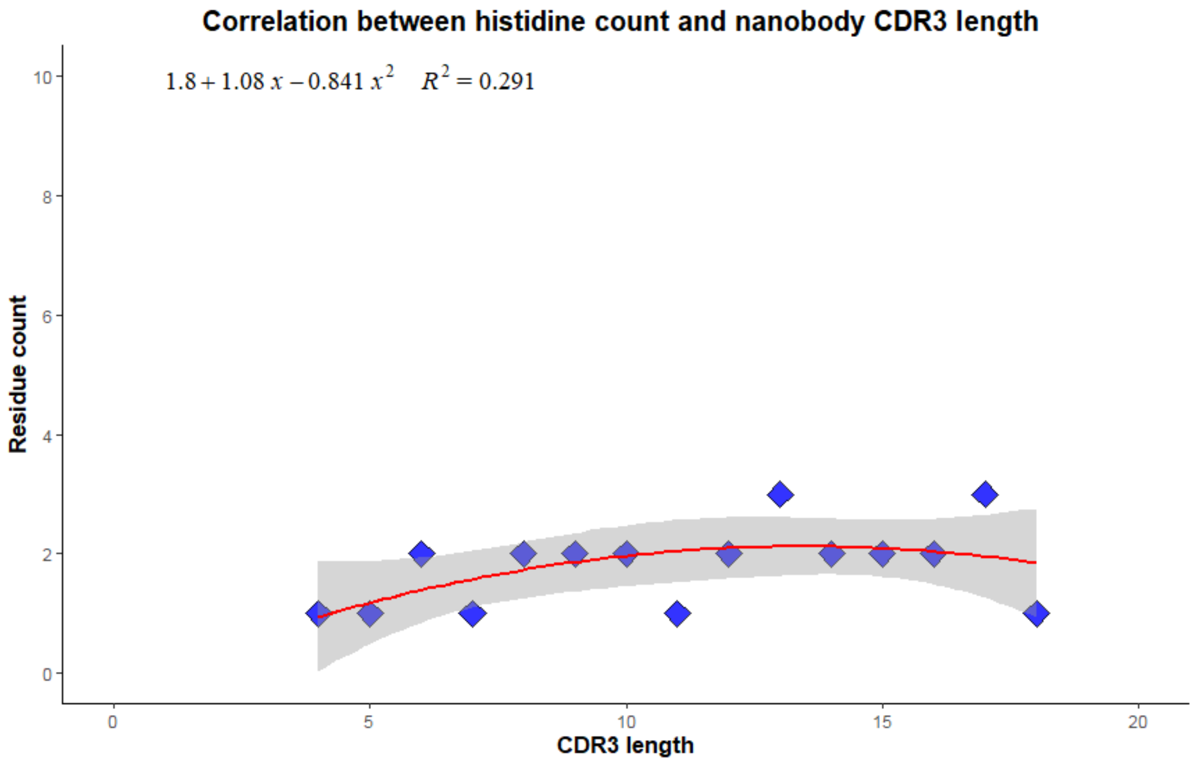

Correlation between histidine count and nanobody CDR3 length
$1.8 + 1.08\,x - 0.841\,x^2 \quad R^2 = 0.291$
Residue count
CDR3 length
0
2
4
6
8
10
0
5
10
15
20

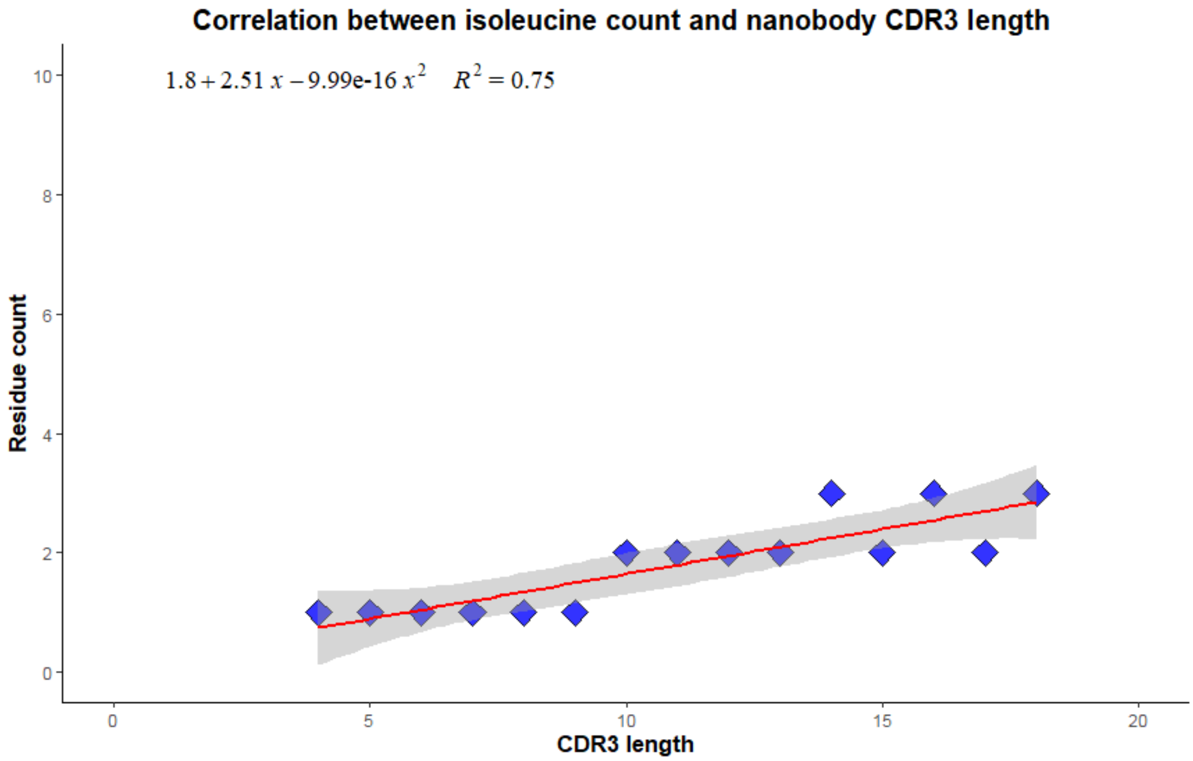

Correlation between isoleucine count and nanobody CDR3 length
$1.8 + 2.51\,x - 9.99\text{e-}16\,x^2 \quad R^2 = 0.75$
Residue count
0
2
4
6
8
10
0
5
10
15
20
CDR3 length

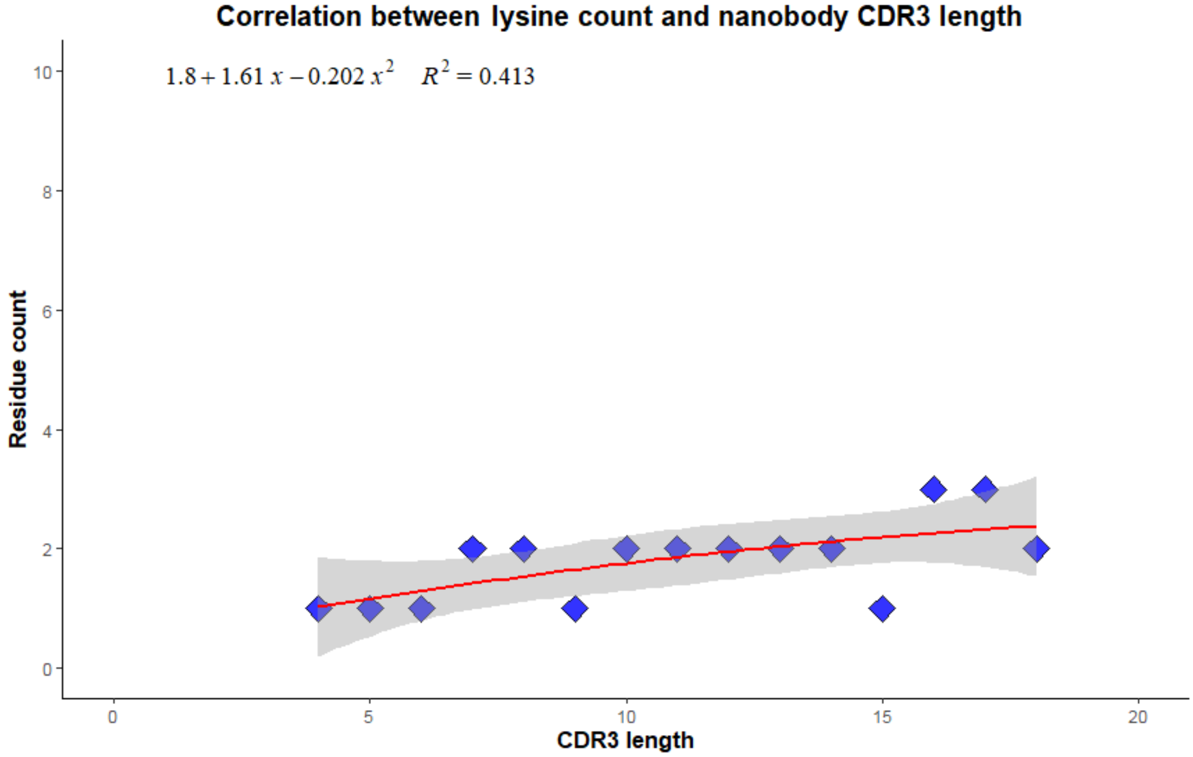

Correlation between lysine count and nanobody CDR3 length
$1.8 + 1.61\,x - 0.202\,x^2 \quad R^2 = 0.413$
Residue count
0
2
4
6
8
10
0
5
10
15
20
CDR3 length

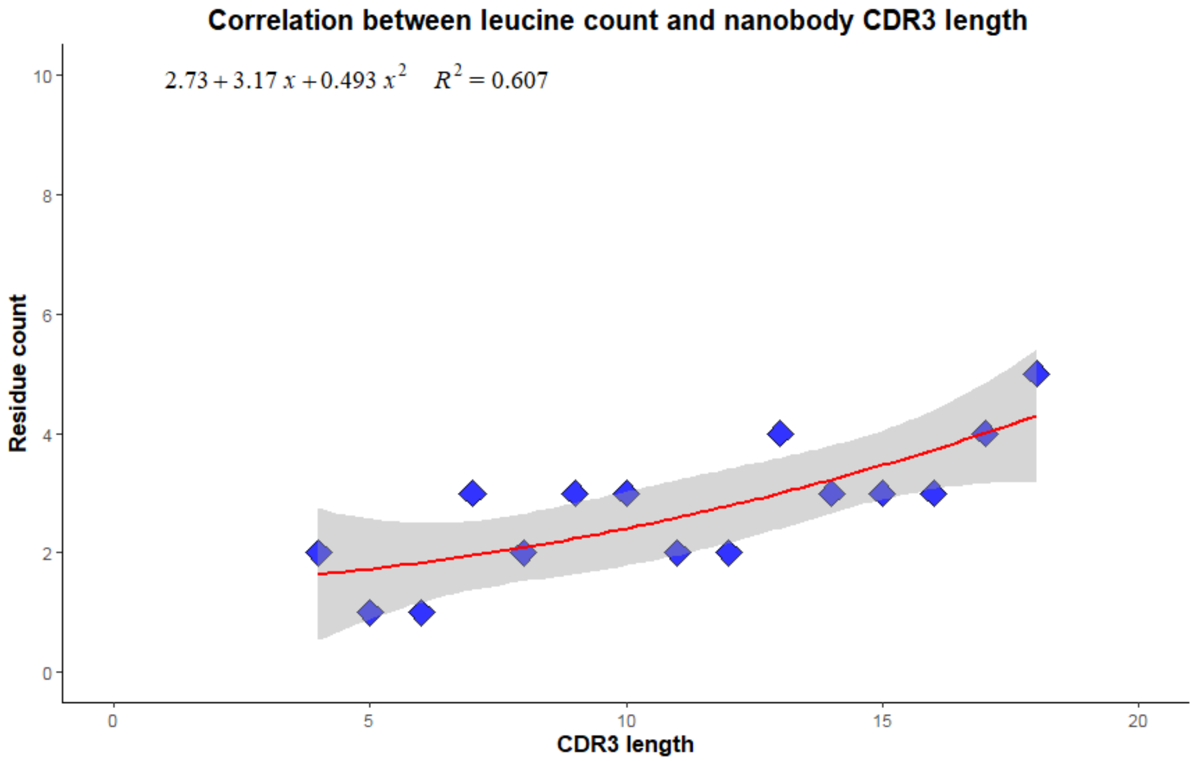

Correlation between leucine count and nanobody CDR3 length
$2.73 + 3.17\,x + 0.493\,x^2 \quad R^2 = 0.607$
Residue count
CDR3 length
0
2
4
6
8
10
0
5
10
15
20

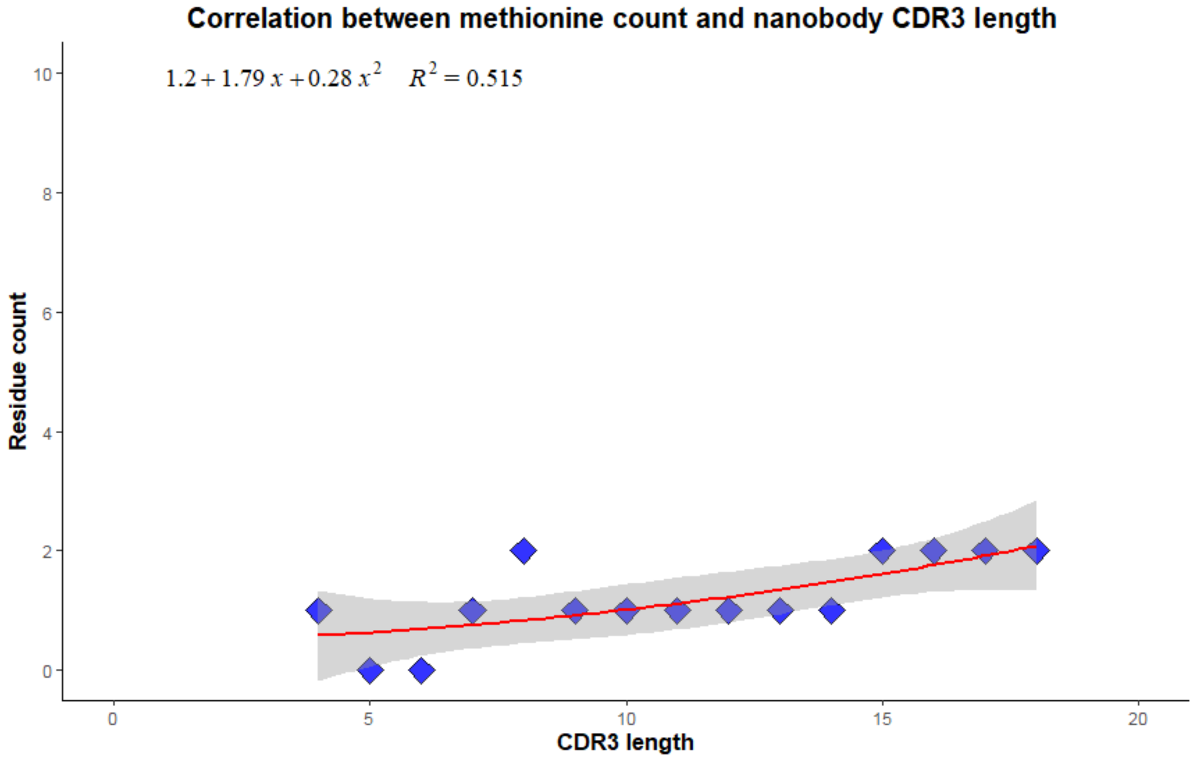

Correlation between methionine count and nanobody CDR3 length
$1.2 + 1.79\,x + 0.28\,x^2 \quad R^2 = 0.515$
Residue count
CDR3 length
0
2
4
6
8
10
0
5
10
15
20

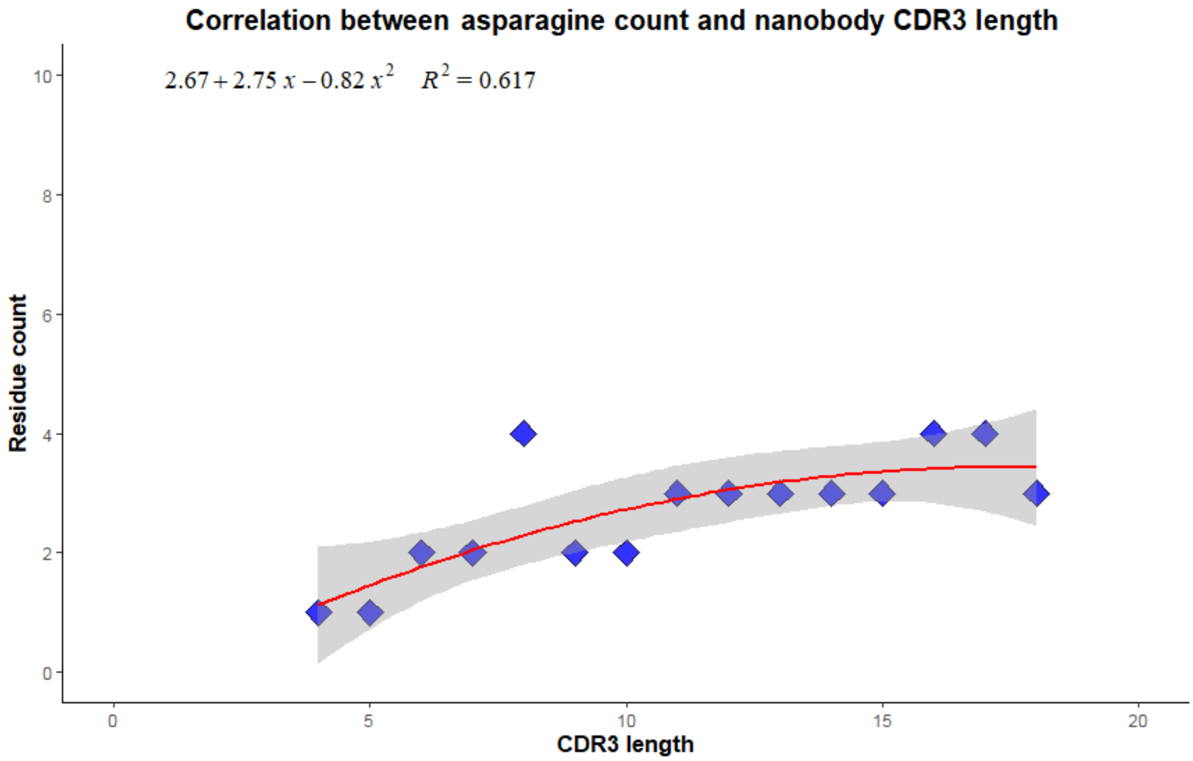

Correlation between asparagine count and nanobody CDR3 length
2.67 + 2.75 x − 0.82 x^2   R^2 = 0.617
Residue count
CDR3 length
0
2
4
6
8
10
0
5
10
15
20

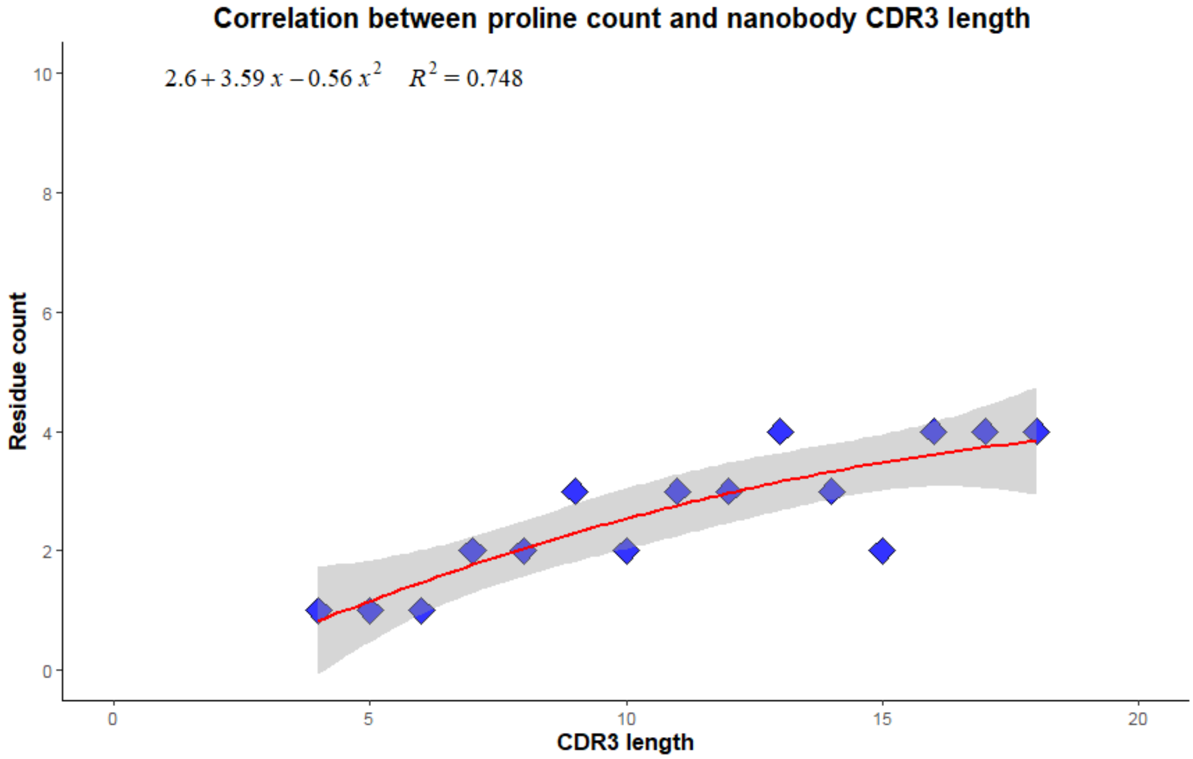

Correlation between proline count and nanobody CDR3 length
2.6 + 3.59 x − 0.56 x^2   R^2 = 0.748
Residue count
CDR3 length
0
2
4
6
8
10
0
5
10
15
20

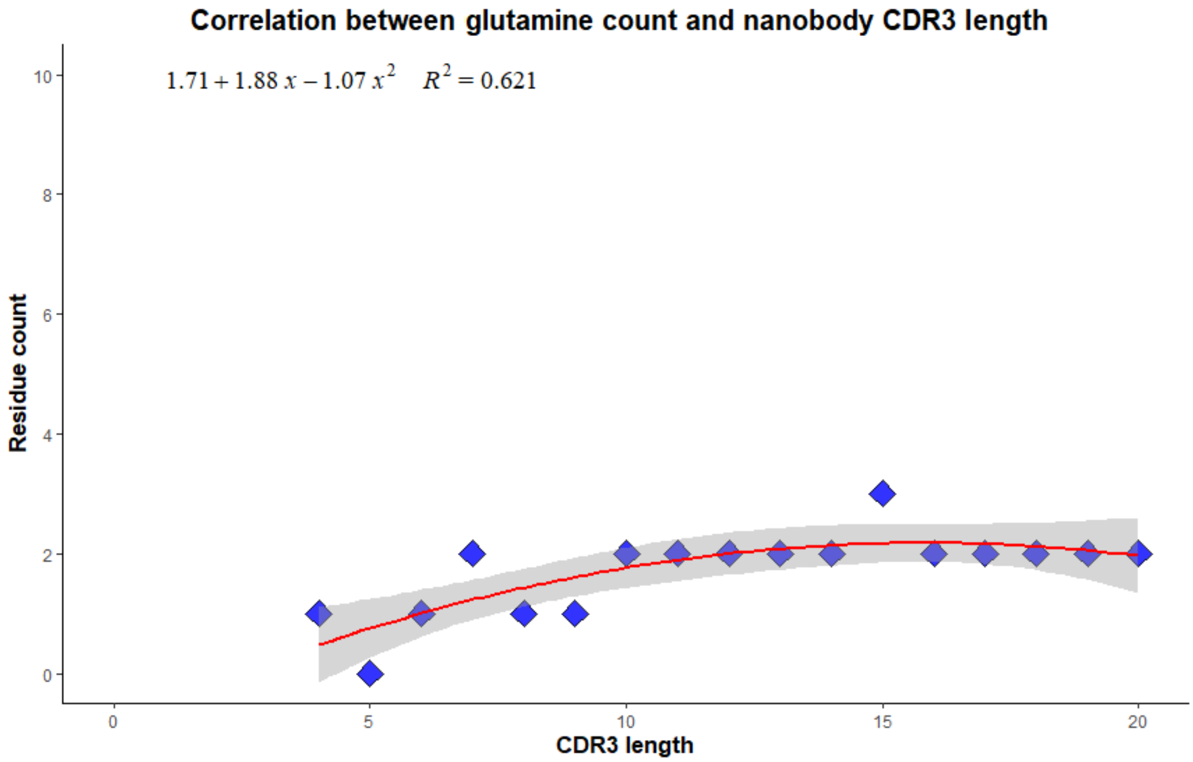
Correlation between glutamine count and nanobody CDR3 length
$1.71 + 1.88\,x - 1.07\,x^2 \quad R^2 = 0.621$
Residue count
CDR3 length
0
2
4
6
8
10
0
5
10
15
20

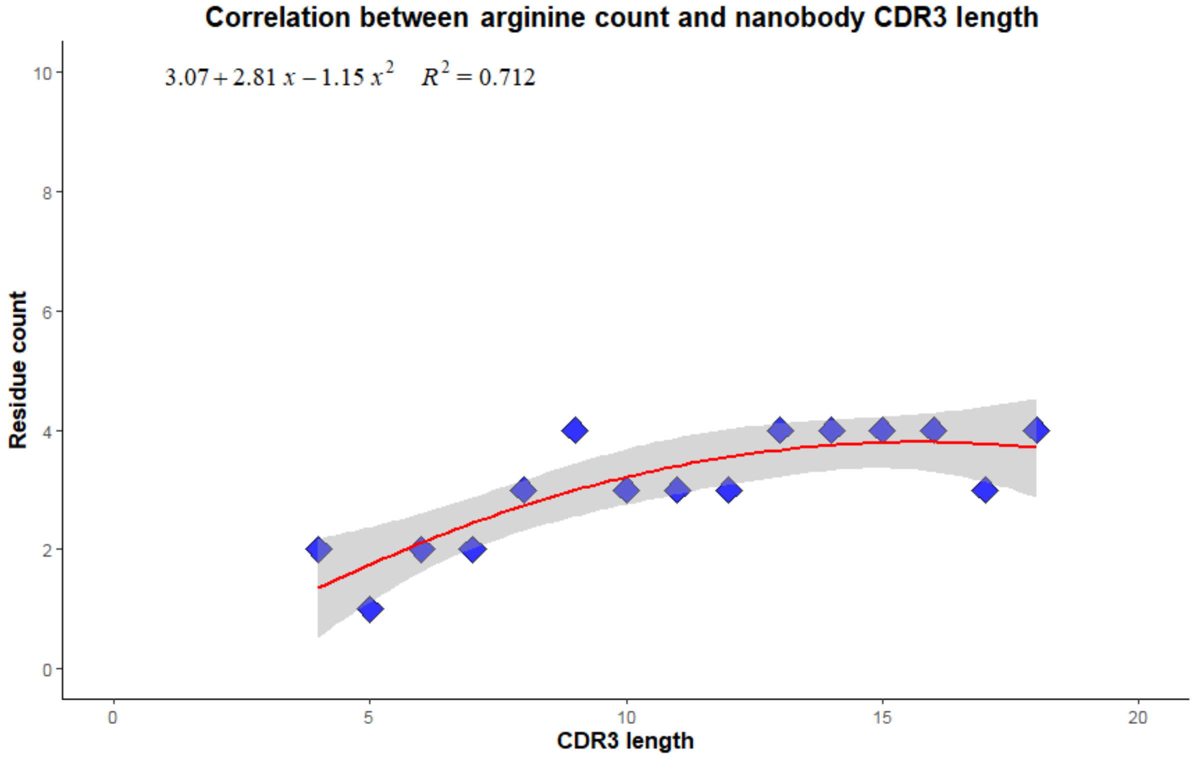
Correlation between arginine count and nanobody CDR3 length
$3.07 + 2.81\,x - 1.15\,x^2 \quad R^2 = 0.712$
Residue count
CDR3 length
0
2
4
6
8
10
0
5
10
15
20

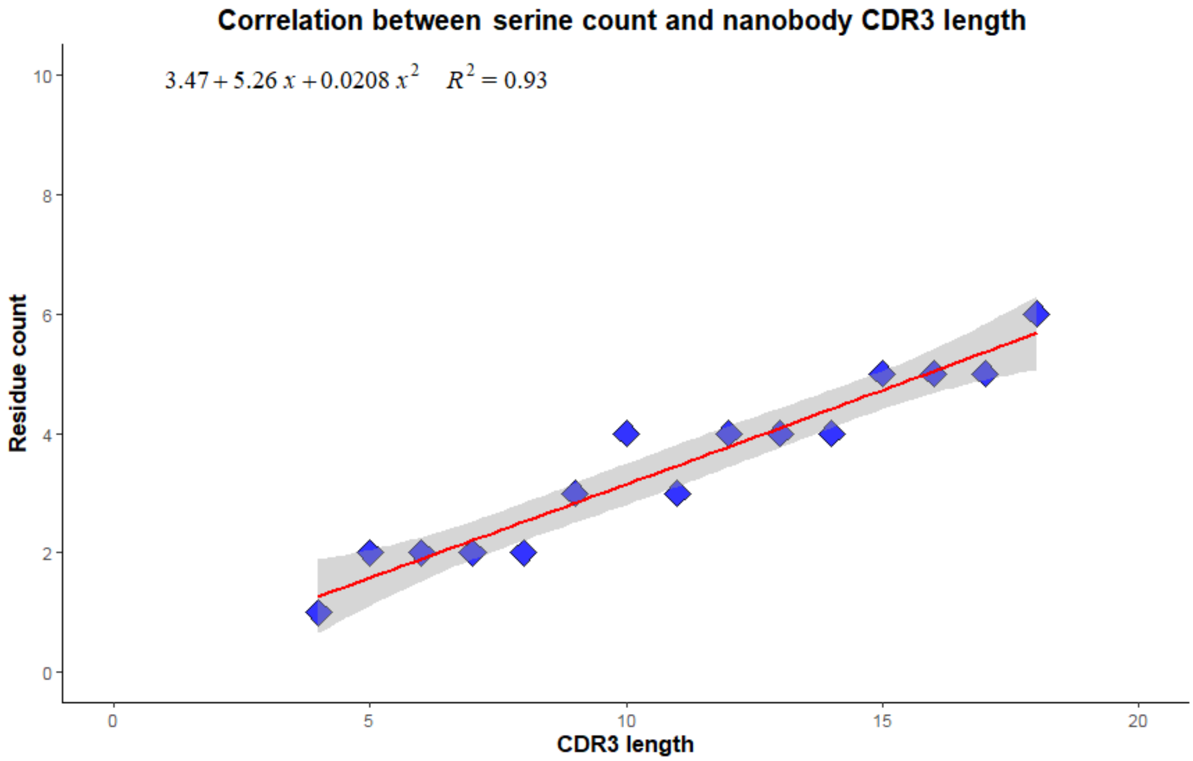

Correlation between serine count and nanobody CDR3 length
$3.47 + 5.26\,x + 0.0208\,x^2 \quad R^2 = 0.93$
Residue count
CDR3 length
0
2
4
6
8
10
0
5
10
15
20

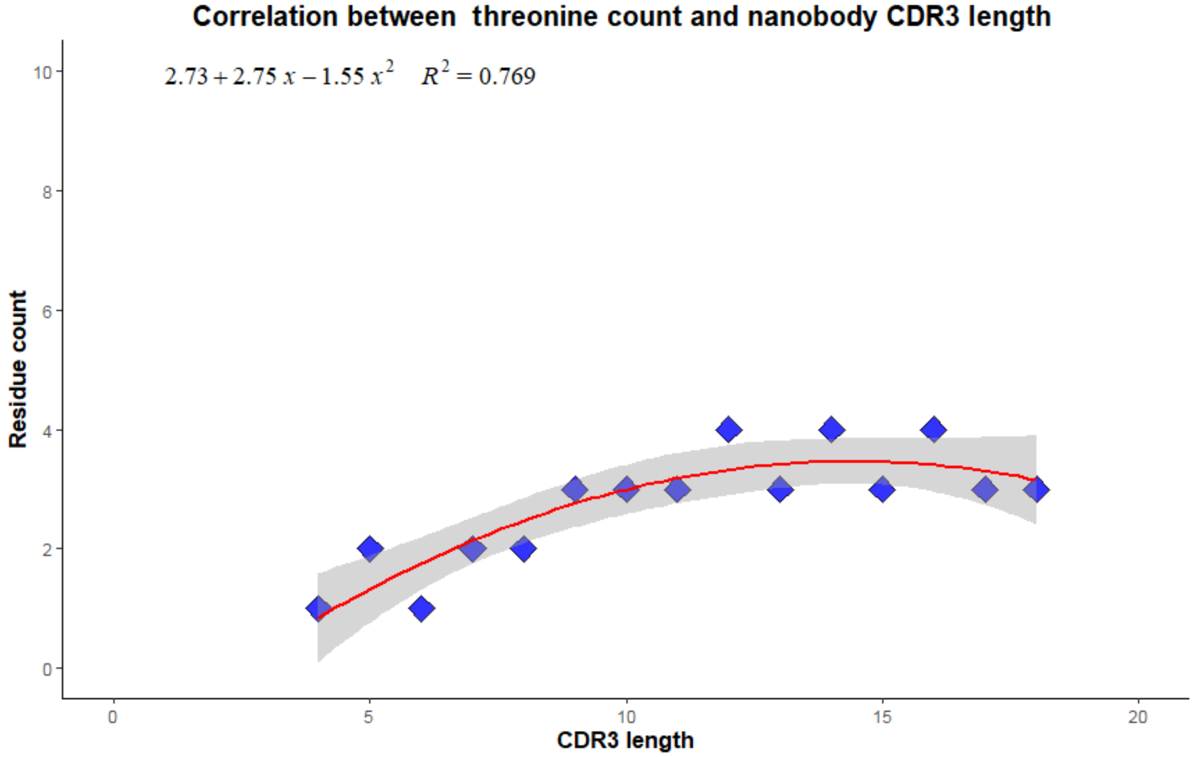

Correlation between threonine count and nanobody CDR3 length
$2.73 + 2.75\,x - 1.55\,x^2 \quad R^2 = 0.769$
Residue count
CDR3 length
0
2
4
6
8
10
0
5
10
15
20

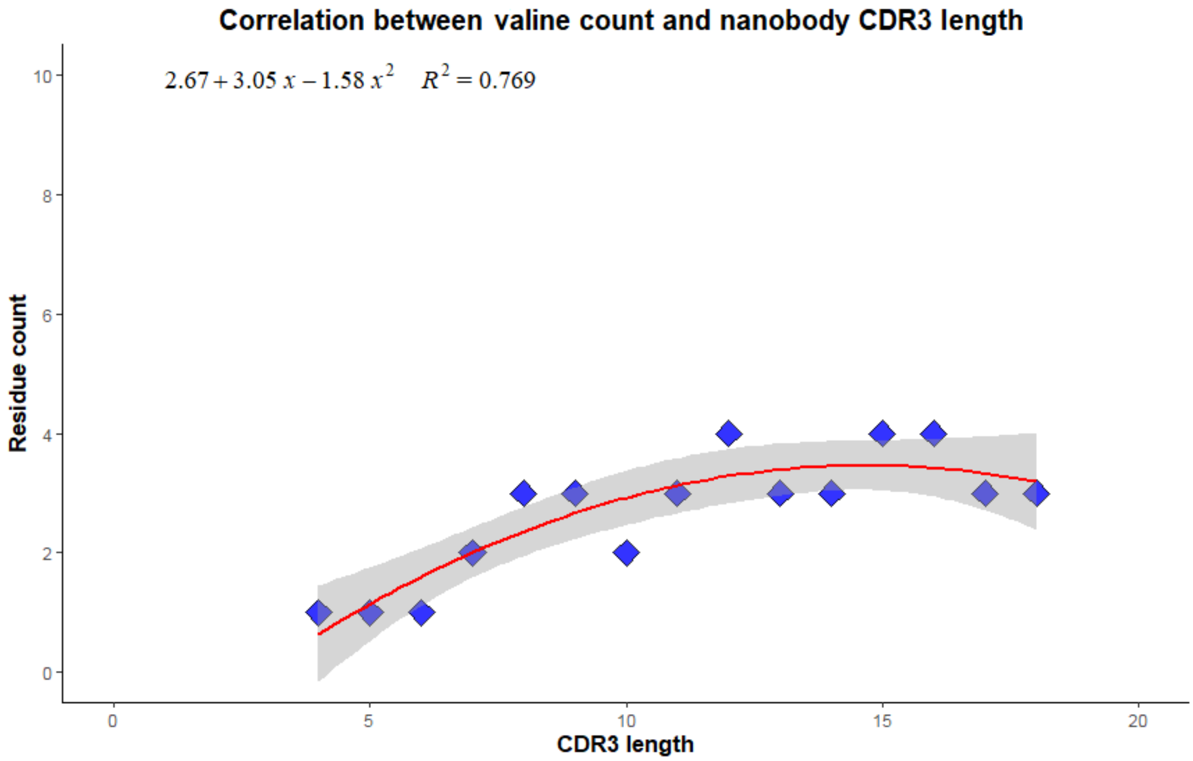

Correlation between valine count and nanobody CDR3 length
2.67 + 3.05 x − 1.58 x²   R² = 0.769
Residue count
CDR3 length
0
2
4
6
8
10
0
5
10
15
20

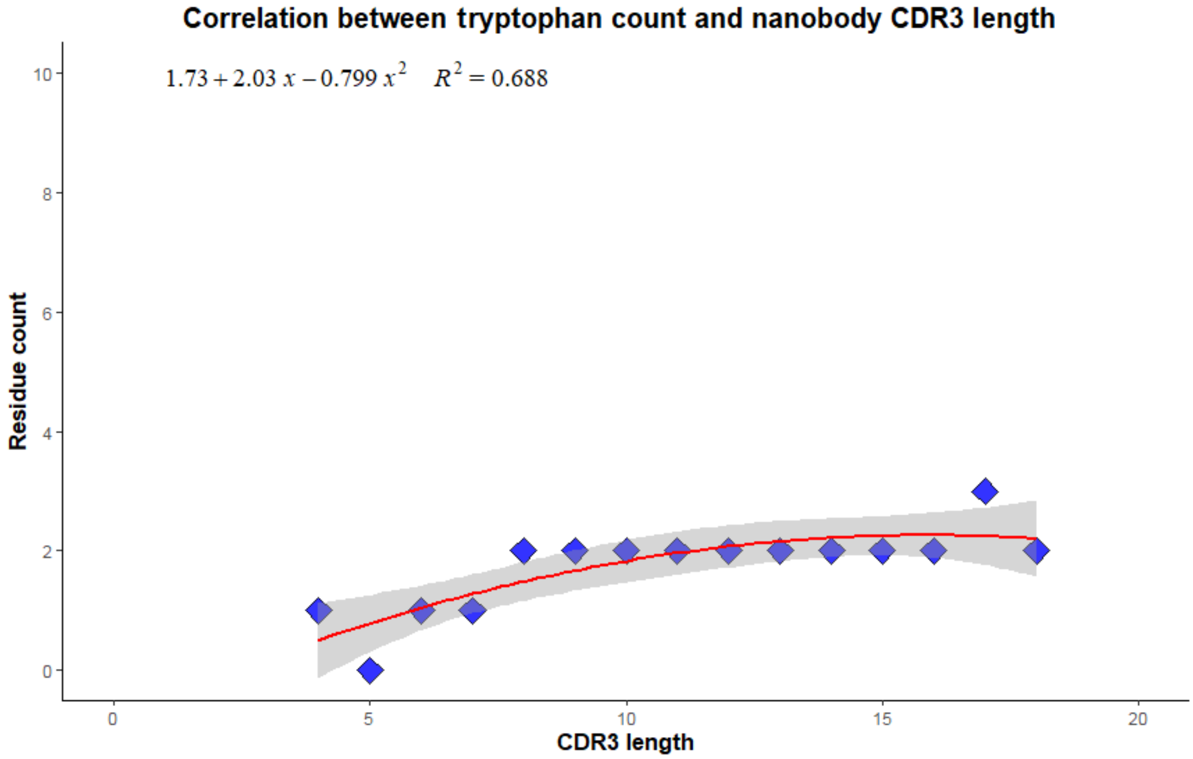

Correlation between tryptophan count and nanobody CDR3 length
1.73 + 2.03 x − 0.799 x²   R² = 0.688
Residue count
CDR3 length
0
2
4
6
8
10
0
5
10
15
20

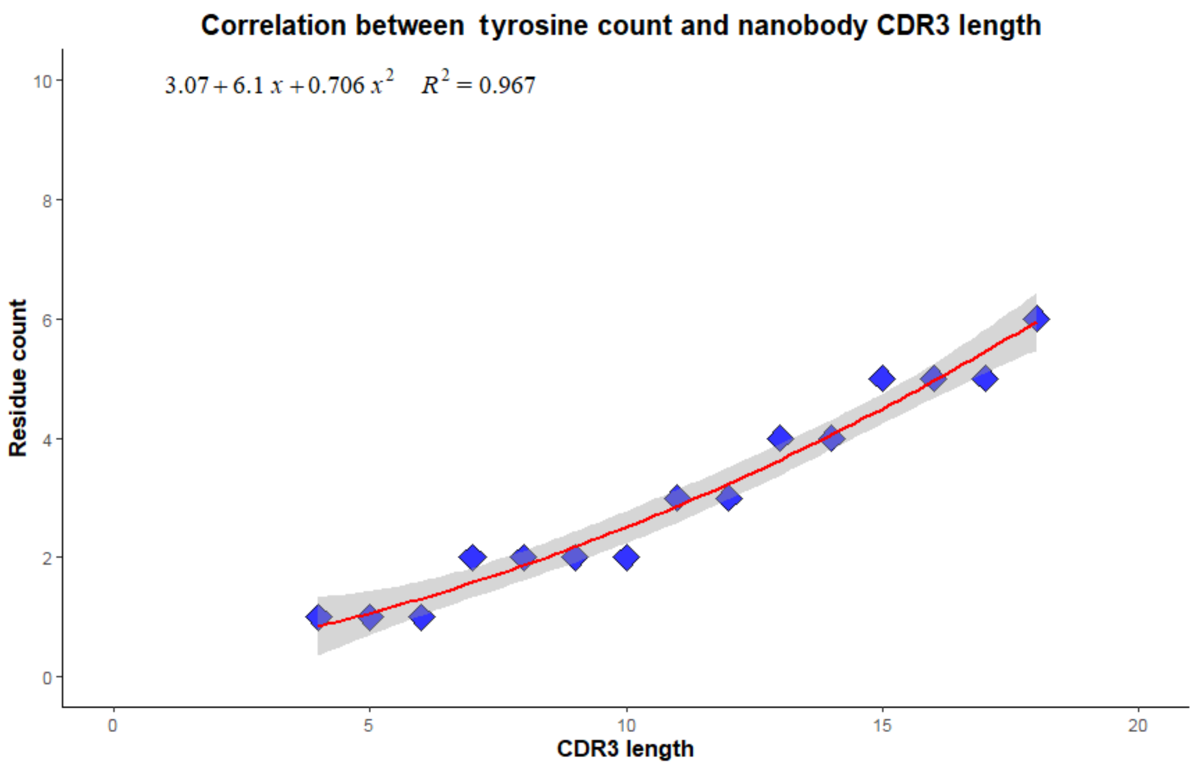

Supplement Figure 2. Correlation between the highest occurrence of amino acids and CDR3 lengths.

Supplement Figure 3

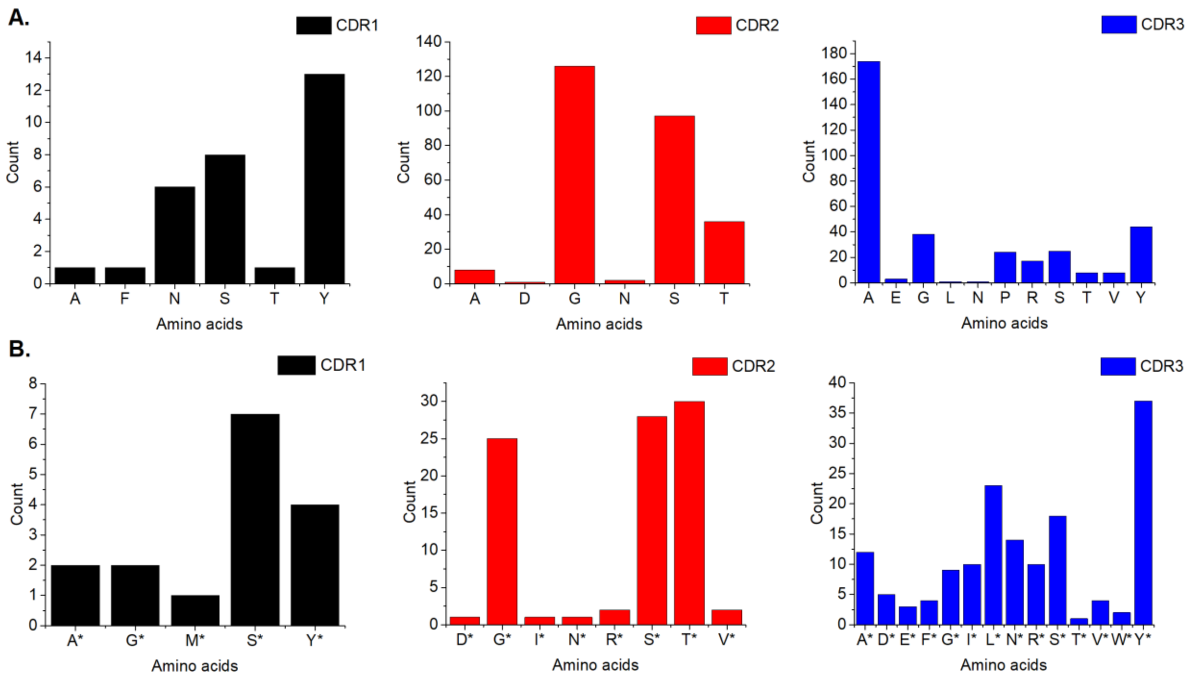

Supplement Figure 3. Occurrence of repeat motifs formed by corresponding amino acids in each CDR region. (A) poly amino acid repeats, (B) oligo repeats.
